# Structure and function of an atypical homodimeric actin capping protein from the malaria parasite

**DOI:** 10.1007/s00018-021-04032-0

**Published:** 2022-02-08

**Authors:** Ábris Ádám Bendes, Petri Kursula, Inari Kursula

**Affiliations:** 1 Biocenter Oulu and Faculty of Biochemistry and Molecular Medicine, University of Oulu, Oulu, Finland; 2 Department of Biomedicine, University of Bergen, Bergen, Norway

**Keywords:** Actin-binding protein, Actin polymerization, Gliding motility, Polymerization kinetics, Regulation, Small-angle X-ray scattering, X-ray crystallography

## Abstract

Apicomplexan parasites, such as *Plasmodium* spp., rely on an unusual actomyosin motor, termed glideosome, for motility and host cell invasion. The actin filaments are maintained by a small set of essential regulators, which provide control over actin dynamics in the different stages of the parasite life cycle. Actin filament capping proteins (CPs) are indispensable heterodimeric regulators of actin dynamics. CPs have been extensively characterized in higher eukaryotes, but their role and functional mechanism in Apicomplexa remain enigmatic. Here, we present the first crystal structure of a homodimeric CP from the malaria parasite and compare the homo- and heterodimeric CP structures in detail. Despite retaining several characteristics of a canonical CP, the homodimeric *Plasmodium berghei* (*Pb*)CP exhibits crucial differences to the canonical heterodimers. Both homo- and heterodimeric *Pb*CPs regulate actin dynamics in an atypical manner, facilitating rapid turnover of parasite actin, without affecting its critical concentration. Homo- and heterodimeric *Pb*CPs show partially redundant activities, possibly to rescue actin filament capping in life cycle stages where the β-subunit is downregulated. Our data suggest that the homodimeric *Pb*CP also influences actin kinetics by recruiting lateral actin dimers. This unusual function could arise from the absence of a β-subunit, as the asymmetric *Pb*CP homodimer lacks structural elements essential for canonical barbed end interactions suggesting a novel CP binding mode. These findings will facilitate further studies aimed at elucidating the precise actin filament capping mechanism in *Plasmodium*.

**Supplementary Information:**

The online version contains supplementary material available at 10.1007/s00018-021-04032-0.

## Introduction

Alongside other members of the vast phylum of Apicomplexa, the unicellular parasites responsible for malaria (*Plasmodium* spp.) use a special actomyosin motor, the glideosome, for motility and host cell invasion during their complex life cycle [1]. Unlike other Apicomplexa, *Plasmodium* parasites encode two non-canonical actin isoforms [2, 3]. In *Plasmodium falciparum*, the causative agent of the most deadly form of malaria in humans, actin isoform I (*Pf*ActI) is constitutively expressed, encompassing the complete parasite life cycle [4]. With < 80% sequence identity with actins from opisthokonts [3, 5], *Pf*ActI polymerizes into short, highly dynamic filaments via the canonical nucleation-elongation mechanism [5, 6]. Fast gliding motility of the parasite requires rapid filament turnover and tight spatial and temporal control. This is conveyed through the innate instability of the *Pf*ActI filaments [7–9] and a modest set of 10–15 actin-binding proteins (ABPs) [10–12]. The *Plasmodium* genome lacks identifiable orthologs of nucleating factors, such as the Arp2/3 branching complex and WASP homologs, the majority of end cappers like gelsolin and tropomodulin, multiple filament-severing and cross-linking agents, and regulators like ENA/VASP proteins [11–13]. The central apicomplexan ABPs include profilin [14], essential for ookinete motility and blood stages [15], nucleation-promoting formins [16], involved in host cell invasion [17] and intracellular replication [18], filament-severing actin-depolymerizing factors [19], vital during the pathogenic erythrocytic stages [20] and implicated in sporogony [21], and actin capping proteins (CPs) [22, 23]. Several of these proteins have atypical or moonlighting properties in comparison to their canonical counterparts [13].

CPs are ubiquitous proteins, usually present in eukaryotes as multiple isoforms [24]. CPs play a crucial role in actin dynamics by binding to the fast-growing barbed end of filamentous actin (F-actin) with high affinity in a Ca^2+^-independent manner [25], limiting protomer exchange to the pointed end. Metazoan CPs characterized to date nucleate polymerization, decrease the elongation rate in preseeded systems, block dilution-induced depolymerization from barbed ends, and increase the critical concentration to the level of the pointed end [24]. CPs are present in various cytoskeletal structures, such as lamellipodial protrusions [26], dynactin [27], and in the sarcomere, linking microfilaments to the Z-disks [28]. CPs are essential for human and zebrafish morphogenesis [29] and belong to the core set of proteins needed to reconstitute actin-based motility in vitro [30]. The average cytosolic concentration of CP in eukaryotes is in the range of 0.5–1.5 µM [31, 32], which, considering the high affinity and 1:1 stoichiometry of CP towards actin filaments, leads to a high number of constantly capped barbed ends in vivo [32]. However, formation of free barbed ends is essential for rapid actin network assembly [33, 34], and control of CP expression levels is required for optimal actin-based cellular functions [30, 31]. Thus, the mechanism of capping/uncapping is modulated by various external factors. Steric and allosteric regulators of CPs include polyphosphoinositides (PIPs), V-1/myotrophin, and CARMIL proteins, while indirect barbed end competitors include formins and ENA/VASP proteins [24].

CPs are comprised of CPα and CPβ subunits, each with a molecular weight of 32–36 kDa [24]. The individual subunits are conserved in most eukaryotes, but the sequence identity between the subunits is typically low [24]. Remarkably, CPα and CPβ share a strikingly similar fold, resulting in a quaternary structure where the two subunits take up a compact arrangement with a pseudo twofold symmetry [35]. Even though subunits and isoforms are expressed at different levels during different stages and cell types [36], the individual subunits are largely insoluble and non-functional when expressed alone in vitro, indicating that they only exist as heterodimers [37].

All currently available structures of heterodimeric CPs have a characteristic shape of a stipitate mushroom [27, 35, 38]. Three N-terminal antiparallel helices form the “stalk” domain flanked by a β-stranded “globule” domain of each monomer. The “cap” is comprised of a well-ordered arrangement of two 5-stranded antiparallel β-sheets, crested by a backbone of four helices formed conjunctly by the subunits. Emerging from the cap structure are the C-terminal “tentacles” of each subunit (termed α- and β-tentacle, respectively) containing a longer flexible loop region and an amphipathic helix [35]. The barbed-end binding of canonical CPs is largely reliant on an electrostatic interaction between a positively charged basic patch of the CP and a negatively charged acidic patch of the last two actin protomers of the filament [39–41]. The basic patch is mainly formed by a localized group of residues on the cap and the α-tentacle. The β-tentacle locks the complex by binding to the hydrophobic pocket of the last actin subunit. However, its main role is to exclude other regulators from binding to this pocket, thus regulating branched actin network assembly [41].

Contrary to the majority of eukaryotes, *Plasmodium* spp. encode only one isoform of each CP subunit [42]. Individual *Pb*CP subunits have evolved distinct biological functions. The heterodimeric *Pb*CPαβ is expressed in the motile extracellular zoite and ookinete stages and is able to reduce the average filament length of heterologous non-muscle actin (β-actin) in vitro [22]. *Pb*CPαβ also readily binds to homologous *Pf*ActI filaments reducing their pelletable form [43]. *Plasmodium knowlesi* CPαβ caps heterologous skeletal muscle actin (α-actin) in vitro in a Ca^2+^-independent, but PIP_2_-dependent manner, thus reducing filament length [44]. Disparate from the other subunit, *Pb*CPβ is upregulated in the insect vector stages and is essential for the locomotion and salivary gland invasion of the highly motile sporozoite stages [22], possibly in its heterodimeric *Pb*CPαβ form [23]. *Pb*CPα, on the other hand, is moderately upregulated in the blood-stage schizonts [23], in which *Pb*CPβ is phenotypically silent. *Pb*CPα is essential for asexual blood stage replication, and loss of it could be rescued by the cognate *Pf*CPα in blood stages, but not in the insect host or in the absence of the α-tentacle domain [23]. Interestingly, a mixed complementation of the CP subunits by *P. berghei* and *P. falciparum* genes was incapable of alleviating the defective mosquito stage. Only a double complementation of *Pf*CPαβ was able to rescue the phenotype. In vitro, *Pb*CPα forms homodimers (*Pb*CPαα), capable of capping homologous *Pf*ActI [43] and heterologous β-actin filaments also in the absence of the α-tentacle [23]. These results suggest that the insect cell stages are regulated by the heterodimeric form, while the pathogenic blood stages are governed by *Pb*CPα, perhaps in a homodimeric form [23, 43].

Here, we present the first crystal structure of a homodimeric CP from the rodent malaria parasite *P. berghei*. While retaining some similarities, the structure demonstrates critical differences compared to canonical heterodimeric CPs. We complement our structural findings with biochemical characterization, showing that the homo- and heterodimers have distinct functions that differ from the canonical CP heterodimer (CapZαβ) and are specific to the *Plasmodium* actin filaments.

## Results

### Crystal structure of the *Pb*CPαα homodimer

**Fig. 1:**
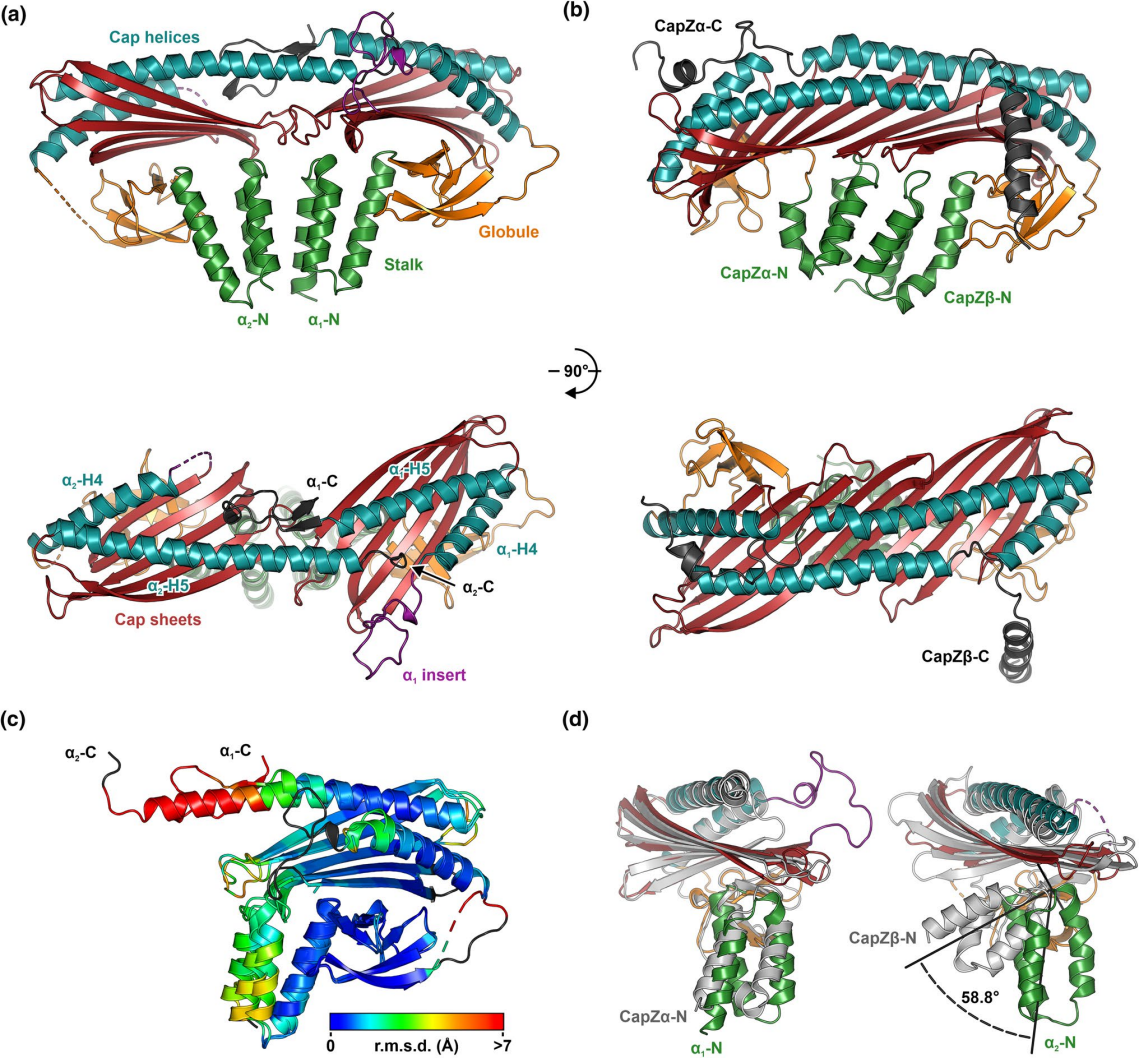
Crystal structure and domain arrangement of *Pb*CPαα^ΔC20^. **a** Crystal structure of *Pb*CPαα^ΔC20^ with the individual domains labeled and depicted in different colors. **b** Crystal structure of CapZαβ (PDB ID: 1IZN [35]) with its domains depicted as for *Pb*CPαα^ΔC20^. **c** Superposition of the *Pb*CPα_1_^ΔC20^ and *Pb*CPα_2_^ΔC20^ subunits. The rainbow color scale represents the r.m.s.d. between the subunits. Residues excluded from the alignment are colored black. **c** Superposition of *Pb*CPα_1_^ΔC20^ to CapZα (left) and *Pb*CPα_2_^ΔC20^ to CapZβ (right). The domains of *Pb*CPαα^ΔC20^ are colored as in panel **a**. CapZαβ (PDB ID: 1IZN) is shown in light gray

To gain structural insight into the unusual homodimerization of *Pb*CPαα, we determined the structure of a C-terminally truncated *Pb*CPαα homodimer (*Pb*CPαα^ΔC20^), which was the only version of the parasite CPs that produced well-diffracting crystals, to 2.2-Å resolution (Fig. 1; Table S1). The two *Pb*CPα^ΔC20^ subunits form a structure that is less compact and intertwined than the canonical heterodimer (Fig. 1a, b), verifying earlier small-angle X-ray scattering (SAXS) and thermal stability analyses [43]. The quaternary structure closely resembles the mushroom shape of metazoan CPs, despite the low sequence conservation (Figs. S1, S2). Albeit being a homodimer, the structure is not completely symmetric. Thus, we will refer to chain A of the crystal structure as *Pb*CPα_1_^ΔC20^ and chain B as *Pb*CPα_2_^ΔC20^. Superposition of the two subunits reveals that structural deviations are mainly present at the dimer interface, possibly to accommodate complementary intersubunit interactions (Fig. 1c). In canonical CP heterodimers, the stalk domain of the β-subunit pivots relative to the core structure, to allow a denser packing. In *Pb*CPα^ΔC20^, the presence of two identical stalk domains disrupts the formation of a compact structure (Fig. 1d). In both canonical heterodimers and the *Pb*CPαα homodimer, the interactions between the stalk domains of the subunits are mainly hydrophobic. In CapZαβ, the helices in the β-subunit are shorter (13, 13, and 8 residues) than in the α-subunit (15, 13, and 20 residues). In the *Pb*CPαα^ΔC20^ homodimer, helices in *Pb*CPα_1_^ΔC20^ are 14, 12, and 16 residues and in *Pb*CPα_2_^ΔC20^ 15, 15, and 16 residues long. Especially the length of H3, which leads to the globule domain, seems to affect the orientation of the entire stalk domain. In addition, there are other factors contributing to the symmetry breaking, discussed below.

*Pb*CPα contains a sequence insertion, not present in metazoan CPs or *Pb*CPβ (Figs. S1, S2). Similar insertions are found in several other apicomplexan ABPs [13]. Their functions are often unknown but could be involved in parasite-specific protein–protein interactions or immune evasion [14, 45]. The 23-residue *Plasmodium* CPα-specific insert is located between the globule and cap β-sheets (Ser136–Ala159). In *Pb*CPα_1_^ΔC20^, this loop protrudes from the structure (Fig. 1a) making space for the long H5 helix (Ile243–Arg281), which would be followed by the α-tentacle in the full-length protein. The base of the insert is stabilized by a partial disulfide bond between Cys158 and Cys179 in the *Pb*CPα_1_^ΔC20^ subunit but not in *Pb*CPα_2_^ΔC20^. *Pb*CPα_2_^ΔC20^ is notably more disordered, due to the reduced number of intersubunit and crystal contacts and has a higher average B-factor than *Pb*CPα_1_^ΔC20^ (Figs. S1, S3), similarly to the crystal structure of CapZαβ [38]. Consequently, the *Plasmodium-*specific insert is not visible in *Pb*CPα_2_^ΔC20^. The H5 helix of *Pb*CPα_1_^ΔC20^ is broken at His267 and turns back towards the same subunit in a β-hairpin-like structure (Leu268-Leu286), instead of extending into *Pb*CPα_2_^ΔC20^, resulting in very different C termini in the subunits (Fig. 1a, c). Interestingly, the first short β-strand of this hairpin motif ends with Ala270, which is the previously identified C-terminal degradation point in full-length *Pb*CPαα [43]. This implies that the C-terminal hairpin-fold of *Pb*CPα_1_^ΔC20^ might be biologically relevant and not an artifact arising from the α-tentacle-truncated *Pb*CPαα^ΔC20^ construct.

### Homodimeric *Pb*CP assembly lacks canonical interaction sites

**Fig. 2:**
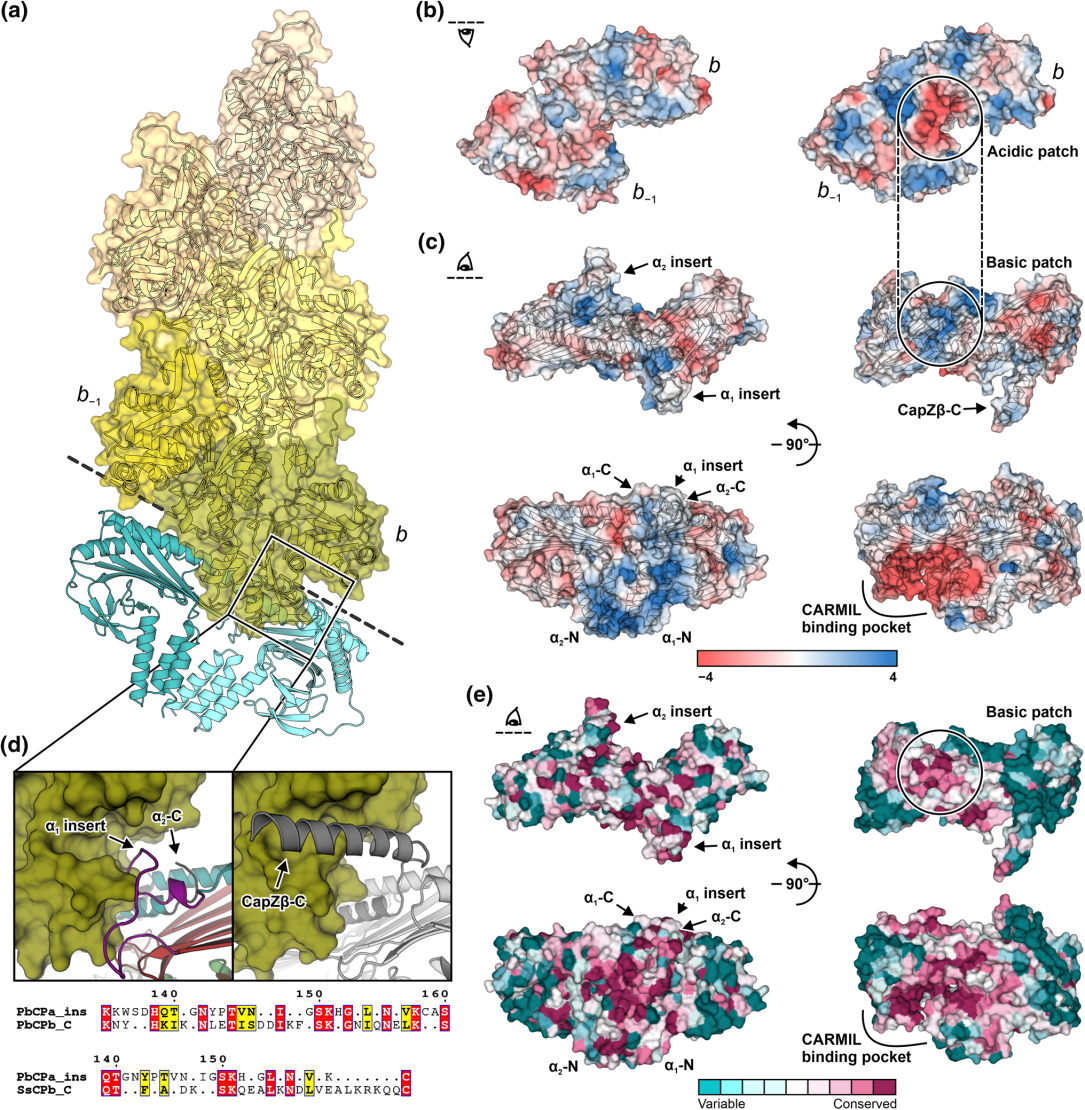
Atypical structural properties of *Pf*ActI and *Pb*CPαα^ΔC20^ suggest a divergent barbed-end binding mode. **a** Model of *Pf*ActI filament (green to beige colored surfaces, PDB ID: 5OGW [7]) capped by *Pb*CPαα^ΔC20^ (blue cartoon) in a canonical arrangement. **b** Electrostatic potential surface of the last (*b*) and penultimate (*b*_−1_) protomer of *Pf*ActI (left) and Arp1 (right, PDB ID: 6F1T [48]) filament barbed ends. **c** Electrostatic potential surface of *Pb*CPαα^ΔC20^ (left) and CapZαβ (right, PDB ID: 6F1T). **d** Hydrophobic pockets of the last subunit of *Pf*ActI (left) and Arp1 (right) filament presented as green surfaces. The *Plasmodium*-specific insert, cap sheets, and cap helices of *Pb*CPαα^ΔC20^ (left) are colored purple, red, and teal, respectively. The CapZαβ (right) α- and β-subunits are shown in light and dark gray, respectively. Sequence alignment (bottom) of the *Plasmodium*-specific insert (PbCPa_ins) with the β-tentacles of *P. berghei* (PbCPb_C: A0A509AQN8) and *S. scrofa* CP (SsCPb_C: A9XFX6), grouped and colored respective to a Risler matrix, using the ESPript convention. **e** Structure of *Pb*CPαα^ΔC20^ (left) and CapZαβ (right) with the surface colored by ConSurf residue conservation scores from low (cyan) to high (magenta)

The observed structural differences result in a non-canonical dimeric CP structure and an example of a rare asymmetric homodimer among proteins in general [46]. It is unclear, however, how these deviations would impact the possible barbed-end binding mode of *Pb*CPαα^ΔC20^. To understand whether a canonical binding mode is compatible with homodimeric CPs, we used molecular docking to generate a model of a *Pb*CPαα^ΔC20^-capped *Pf*ActI filament (Fig. 2a). The expression of Arp1 in *P. berghei* [47] could implicate the existence of *Pb*CP-capped Arp1 filaments in *Plasmodium*, in a similar manner as the metazoan dynactin [27]. Therefore, we used the cryo-EM structure of CapZαβ-capped Arp1 filament in *Sus scrofa* dynactin [48] as a basis for modeling. During the preparation of this manuscript, a high-resolution cryo-EM structure of mammalian β-actin capped with CapZ was published [41]. This has also been used for comparisons to our model (Figs. S4, S5).

The electrostatic potential surfaces at the actin filament barbed end (Fig. 2b) and CPs (Fig. 2c) reveal the absence of canonical charged interaction points in the *Plasmodium* proteins (Fig. S4), even though residues involved in forming these interaction surfaces are conserved (Figs. S1, S2, S6). In *Pf*ActI, flanking residues mask the apparent charge of the patch (Figs. 2b, S4a). Differences in surface electrostatic potential [49] could also play a large role in the divergent nucleation and polymerization properties of *Pf*ActI [6]. Residues of the basic patch in *Pb*CPαα^ΔC20^ are more buried in the structure, not in close proximity to each other, and the slight asymmetry of the homodimer does not compensate significantly for the loss of contributing residues of *Pb*CPβ. The putative basic patch spans a larger area in the *Pb*CPαβ model, only loosely resembling the arrangement in CapZαβ (Fig. S4b). Possibly due to the absence of CARMIL proteins in *Plasmodium* [12], the positively charged CARMIL-binding pocket of canonical CPs [50] is less prevalent in the *Pb*CPαβ model (Fig. S4b) and absent in *Pb*CPαα^ΔC20^ (Fig. 2c). Upon binding the barbed end, the β-tentacle of CapZαβ locks the complex by binding the hydrophobic pocket of the terminal actin subunit [39–41]. In our model, the *Plasmodium*-specific insert of *Pb*CPα_1_^ΔC20^ is close to the expected position of the β-tentacle (Figs. 2d, S5). This insert shares many possible hydrophobic or electrostatic interaction points with the β-tentacle. These residues are mainly conserved across *Plasmodium* spp. (Fig. S1), suggesting a possible role for the insert in barbed-end binding in the absence of the β-tentacle. Mapping per-residue sequence conservation of *Plasmodium* CPα sequences on the structure of *Pb*CPαα^ΔC20^ reveals that core residues, especially those involved in the intersubunit surface, are highly conserved, suggesting that CPαα homodimers likely exist in other *Plasmodium* spp. Canonical binding partner interaction points present in CapZαβ lack localized sequence conservation in *Pb*CPαα^ΔC20^ (Figs. 2e, S1).

Comparing our barbed end model to the CapZαβ-capped β-actin filament [41] shows that the predicted relative orientation of *Pb*CPαα^ΔC20^ is in good agreement with the orientation of CapZ in the experimental structure (Fig. S5). The *Pb*CPα_1_^ΔC20^ subunit is located 5–6 Å closer to the last actin subunits due to the absence of the tentacle domains and the compatible orientation of the *Plasmodium*-specific insert. The N-terminal stalk domains align surprisingly well, while the globule of *Pb*CPα_2_^ΔC20^ is twisted compared to its CapZβ counterpart, as discussed previously. Interestingly, both dynactin- and β-actin-bound CapZαβ structures describe a more pronounced kink of the H5 helix, just before the α-tentacle, towards the central β-sheet (Fig. S5c). The β-sheet in the β subunit is also slightly distorted to adjust for the H5 helix orientation. These differences stem possibly from the conformational changes required for barbed-end binding [51, 52]. The unique β-hairpin structure in *Pb*CPα_1_^ΔC20^ corresponds well to the location of this distortion (Fig. S5d) and could potentially serve a similar function.

The differences in the CARMIL-binding pocket instigated us to take a deeper look into other CP regulators in *Plasmodium*. While the majority of these are absent from *Plasmodium* [12], we cannot exclude the possibility of other, so far uncharacterized, regulators. Our search for V-1/myotrophin homologs and the CP-binding and uncapping motif of CARMIL proteins among known *Plasmodium* transcripts, or for the S100B interaction motif in *Plasmodium* CPs resulted in no clear hits. To date, PIP_2_ is the only described direct CP regulator in *P. knowlesi* with HSC70 mentioned as a binding partner uninvolved in the capping activity [44]. Residues involved in canonical interactions with protein regulators are generally not conserved in *Plasmodium* CPs (Figs. S1, S2), with the exception of several basic residues (Lys278 and Lys282 in *Pb*CPα, Arg233 in *Pb*CPβ), which are involved in canonical F-actin interaction and PIP_2_ binding [39, 40, 51].

### Conservation of the CP domain structure

Canonical CP subunits share a strikingly similar fold, despite low sequence identity. Compared to the heterodimeric CPs, the homodimeric *Pb*CPαα^ΔC20^ has an increased surface area but a significantly decreased dimer interface area (Table S2). Expectedly, the canonical CP heterodimers are more similar to each other than to the *Plasmodium* CP homodimer (Table S3). Despite the low sequence identity, both *Pb*CPαα^ΔC20^ subunits are strikingly similar to canonical CPα isoforms (Table S4). This confirms our previous findings [43] and is further substantiated by the identifiable hits of CP-related CATH domains (1.20.1290.20 and 2.40.160.80) in the *Pb*CPαα^ΔC20^ structure. The identified domains in *Pb*CPαα^ΔC20^ superimpose well with canonical CP subunits (Table S5), suggesting the importance of conserved structural elements in the actin filament capping function among broad taxa, despite the lack of sequence conservation. Indeed, during the erythrocytic stages of *P. berghei*, where *Pb*CPβ is phenotypically silent and homodimeric *Pb*CPαα takes over functionally, *Pb*CPα can be complemented by its *Pf*CPα cognate despite the fact that they only share 52% sequence identity [23].

### *Plasmodium* CPs form similar-shaped homo- and heterodimers in solution

Our previous SAXS studies [43] agree well with the crystal structure suggesting that the homodimeric structure is a native conformation and not an artifact resulting from crystal packing or the absence of the α-tentacle domains. To gain further insight into the homo- and heterodimerization of *Pb*CPs, we carried out further SAXS and homology modeling studies based on the crystal structure of *Pb*CPαα^ΔC20^.

**Fig. 3:**
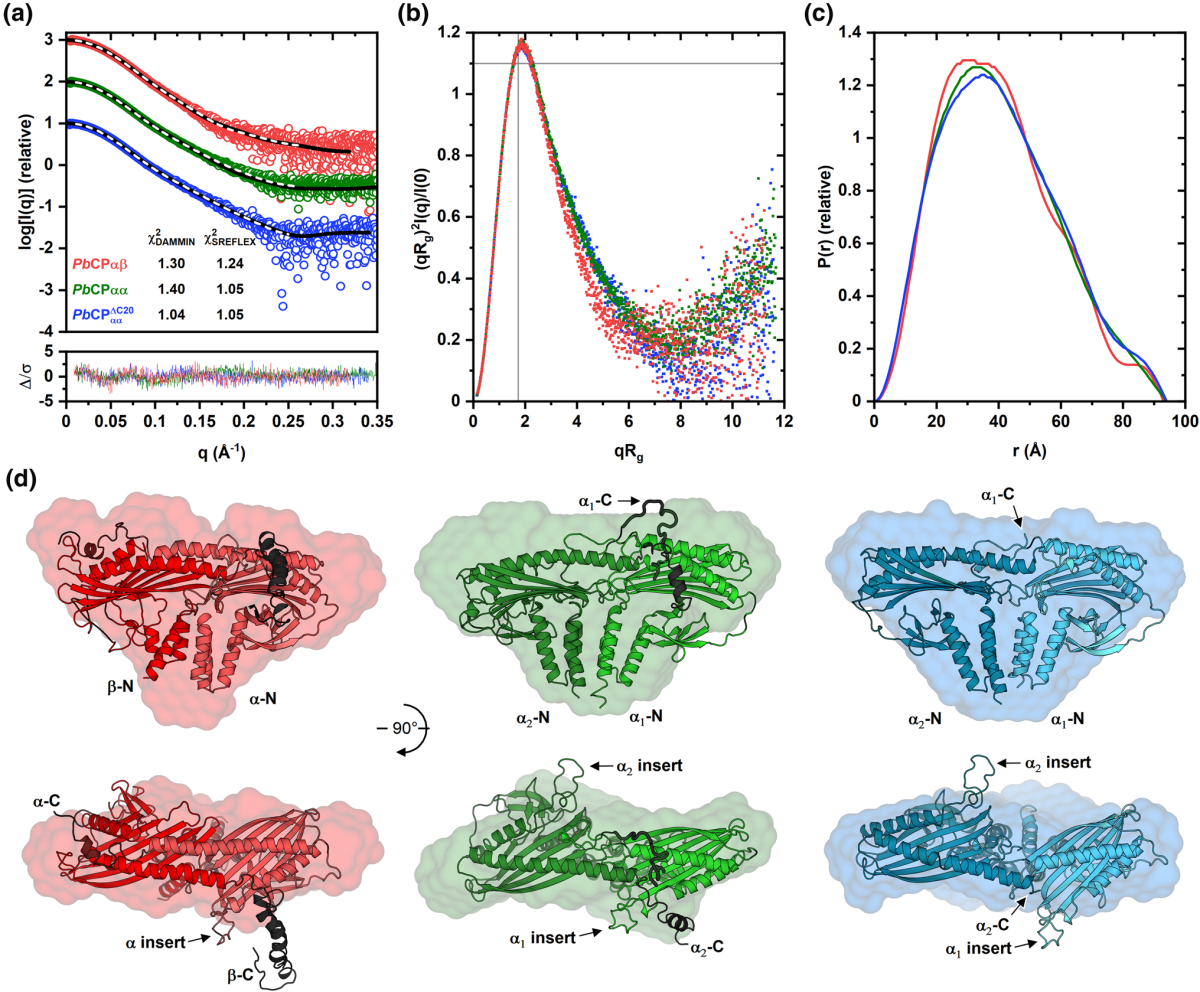
*Pb*CP homo- and heterodimers have a canonical shape in solution. **a** Experimental scattering curves of *Pb*CPαβ, *Pb*CPαα, and *Pb*CPαα^ΔC20^ (red, green, and blue open circles, respectively) and the respective fits of DAMMIN (white dashed lines) and SREFLEX (black line) models to the data. The *χ*^2^ values of the different models to the data are indicated. Weighted residuals of the SREFLEX model fits are denoted in the lower graph (lines colored respectively), where $\Delta /\sigma = \left[ {I_{\exp } \left( q \right) - cI_{\bmod } \left( q \right)} \right]/\sigma \left( q \right)$. **b** Dimensionless Kratky plot (colored similar to **a**). **c** Real-space distance distribution plot (colored similar to panel A). **d** Cartoon models of *Pb*CPs (colored as in **a**, with chain A in lighter color) and the corresponding SAXS envelopes as surface representations. The tentacle domains of each structure are colored black. Experimental data for *Pb*CPαα^ΔC20^ for comparison were used from a previous publication [43]

*Pb*CPαα^ΔC20^, *Pb*CPαα, and *Pb*CPαβ all form similar pseudo-symmetric dimers in solution (Fig. 3). The SAXS data indicate folded, globular proteins containing flexible parts, with *Pb*CPαβ being somewhat more compact than the homodimers (Fig. 3a, b). Interparticle distances are similar for all *Pb*CPs, with the heterodimer showing slight bimodality (Fig. 3c). Modeling into the SAXS data suggests that *Pb*CPαβ forms a more canonical tight structure, whereas both subunits of the homodimers adopt a loose arrangement, similar to the *Pb*CPαα^ΔC20^ crystal structure (Fig. 3d). The orientation of the *Plasmodium*-specific insert and the tentacle domains of both subunits cannot be reliably deduced from the SAXS data, indicating that they are disordered in solution, which is characteristic of the tentacle domains in canonical CPs as well [35, 38, 53].

### *Pb*CPs control actin polymerization in a non-canonical manner

**Fig. 4:**
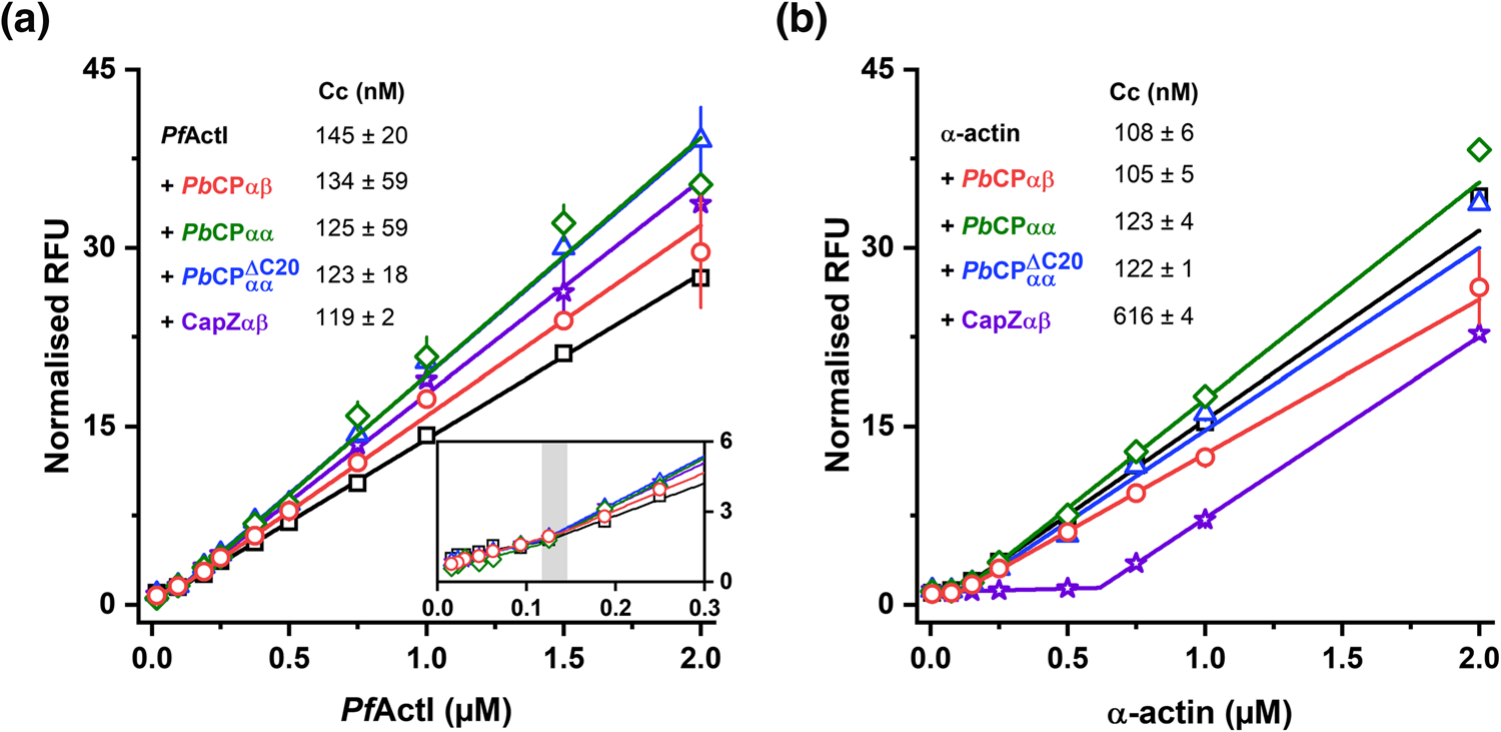
*Pb*CPs do not affect the critical concentration of actin polymerization. **a** *Pf*ActI filaments (open black squares) capped by *Pb*CPαβ, *Pb*CPαα, *Pb*CPαα^ΔC20^, and CapZαβ (open red circles, green diamonds, blue triangles, and purple stars, respectively). The gray shading indicates the range of determined Cc_app_ from two-line fits (respectively colored lines). **b** α-actin filaments (open black squares) capped by *Pb*CPαβ, *Pb*CPαα, *Pb*CPαα^ΔC20^, and CapZαβ (as in **a**). Errors represent SD (*n* = 3). *RFU* relative fluorescent unit, normalized to the lowest concentration

CPs typically block the barbed end, increasing its apparent critical concentration (Cc_app_) to that of the pointed end [24]. *Pb*CPs increase the supernatant fraction of polymerized *Pf*ActI in pelleting assays [43], which could be explained either by filament shortening or by limited depolymerization due to the increase of Cc_app_. However, in dilution series of pyrene-labeled *Pf*ActI filaments, none of the *Pb*CPs affected the fluorescence signal of *Pf*ActI, even at high stoichiometric ratios (Fig. 4a). Similar behavior was observed with heterologous α-actin (Fig. 4b), despite *Pb*CPs being able to modulate α-actin [44] or β-actin polymerization [22, 23]. Furthermore, gelsolin, which is a major barbed end capper [54] absent from *Plasmodium* spp. [11], does not affect the Cc_app_ of *Pf*ActI filaments (Fig. S7) or *Pf*ActI depolymerization dynamics [6]. The decreased amount of pelletable actin in the presence of *Pb*CPs and the lack of effect on the fluorescence signal in the pyrene assay are likely due to a decrease in filament length to oligomers that do not sediment. Another possibility would be CP binding to the barbed end of actin in either monomeric or dimeric form, thus increasing its fluorescence signal similarly to polymerization.

**Fig. 5:**
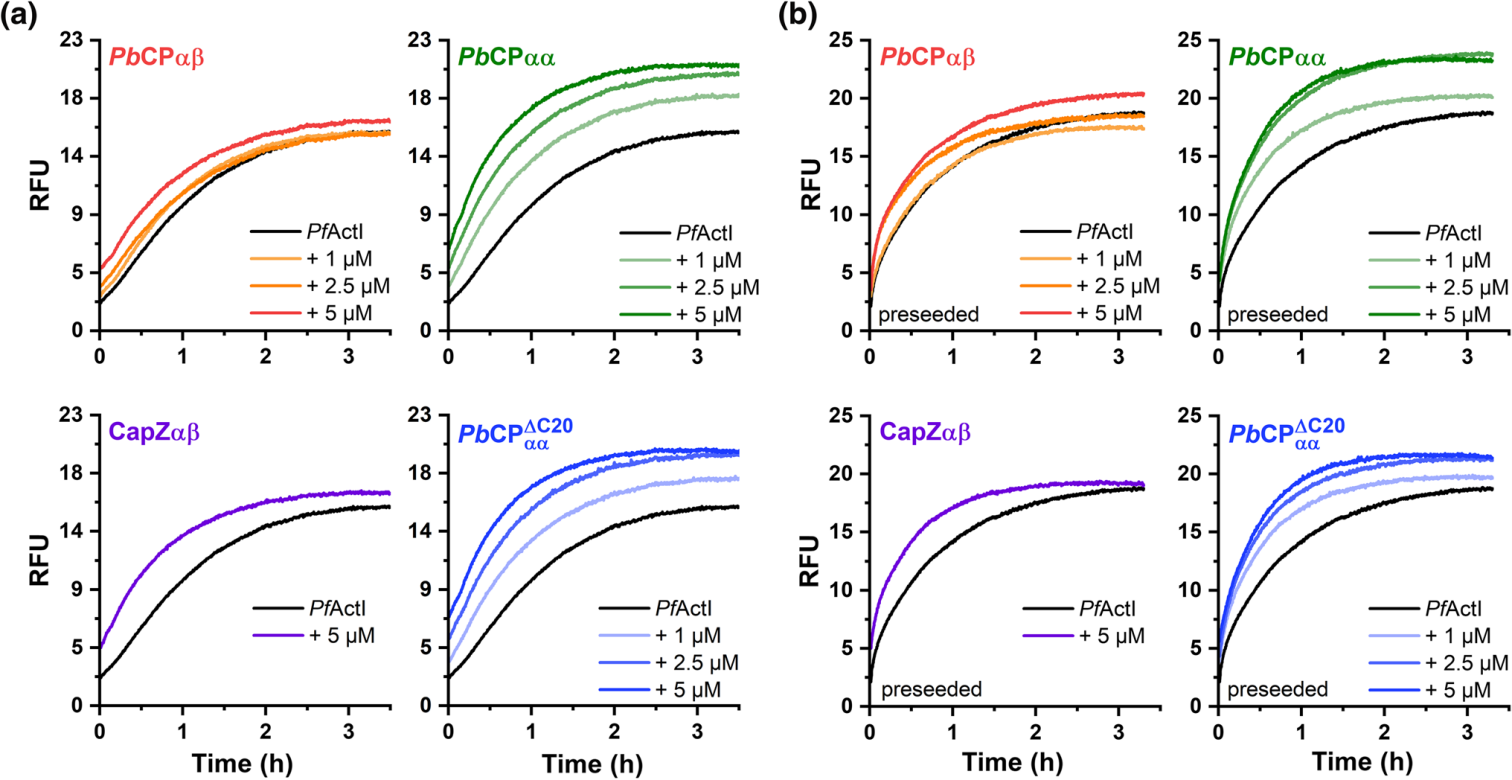
Atypical regulation of *Pf*ActI polymerization kinetics by *Pb*CPs. **a** Polymerization curves of *Pf*ActI in the absence and presence of increasing concentrations of *Pb*CPαβ, *Pb*CPαα, *Pb*CPαα^ΔC20^, and CapZαβ. The *Pf*ActI-only polymerization curve is shared between measurements and the fluorescence levels are comparable. **b** Polymerization of *Pf*ActI on capped, preformed homologous filaments in the absence and presence of increasing concentrations of *Pb*CPαβ, *Pb*CPαα, *Pb*CPαα^ΔC20^, and CapZαβ. The *Pf*ActI-only polymerization curve is shared between preseeded measurements and the fluorescence levels are comparable. *RFU* relative fluorescent unit

In higher eukaryotes, CPs nucleate filaments, abolishing the lag phase, but block subunit exchange at the barbed end [24], reducing both the initial elongation velocity and steady-state filament mass. Contrary to expectations, *Pb*CPs increased *Pf*ActI elongation rates and considerably raised the steady-state fluorescence levels, indicating elevated amounts of actin in non-monomeric forms (Fig. 5). The effect of *Pb*CPs on nucleation was ambiguous, due to the nature of *Pf*ActI polymerization curves, which lack a pronounced lag phase [6]. The heterodimeric CPs did not modulate the steady-state fluorescence levels of *Pf*ActI to the extent seen with the *Pb*CP homodimers, which could also be observed as slope differences in the Cc assays (Fig. 4). Interestingly, all *Pb*CPs seem to block the barbed end of α-actin, similarly to CapZαβ (Fig. S8). However, they do not display any notable nucleation activity, and unlike heterodimeric CPs, the *Pb*CP homodimers increase the initial fluorescence levels. *Pb*CPαα^ΔC20^ displays an only slightly diminished capping effect, despite the absence of the tentacle domains [39, 40]. Despite its in vivo indispensability [23] the reduced importance of the *Plasmodium* α-tentacle domain in *Pb*CPs has been suggested before [43].

**Fig. 6:**
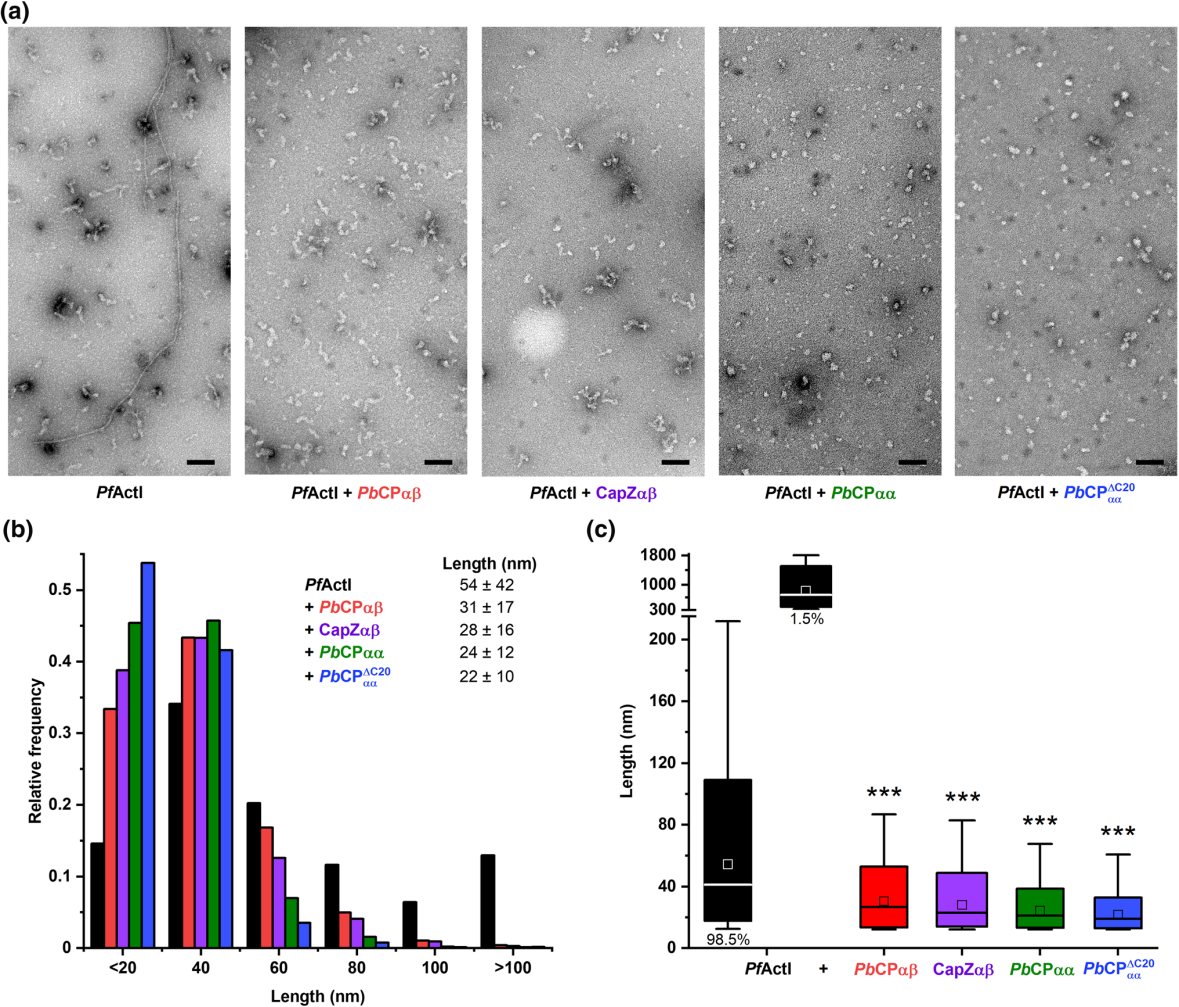
CPs remove long *Pf*ActI filaments and reduce average filament length. **a** Representative negative stained EM micrographs of 1 µM *Pf*ActI polymerized alone or together with 1.2 µM CPs. Scale bars represent 100 nm. **b** Frequency distribution of the measured filament particles of polymerized *Pf*ActI alone (black, *n* = 1930) or with *Pb*CPαβ (red, *n* = 3305), CapZαβ (purple, *n* = 2108), *Pb*CPαα (green, *n* = 3883), and *Pb*CPαα^ΔC20^ (blue, *n* = 2251). Filaments larger than 300 nm are excluded from the distribution plot. Inset shows the calculated means of the filament lengths. Errors represent SD. **c** Box plot representation of the filament distributions for *Pf*ActI alone or capped with CPs. *Pf*ActI filaments longer than 300 nm are displayed separately. Boxes encompass 80% of the distribution around the median (horizontal bars) and whiskers extend to the 1st and 99th percentiles. The mean values are shown as squares. Asterisks represent significant difference (***p < 0.001) between the medians when compared against the smaller *Pf*ActI species (98.5% of measured *Pf*ActI filaments) using Mann–Whitney tests

Major differences between the homo- and heterodimers are seen in the steady-state *Pf*ActI fluorescence levels (Fig. 5a). We also measured length distributions of *Pf*ActI filaments polymerized with and without homo- and heterodimeric CPs from negative-stained electron micrographs (Fig. 6). *Pf*ActI alone forms mainly short, non-helical structures with a sporadic presence of long helical filaments [8]. Upon incubation with CPs, the long filaments were completely removed (Fig. 6a) and the shorter species became more abundant. The average filament length is reduced by half (Fig. 6b, c) as also seen for canonical actins [22, 23, 44]. In line with the polymerization assays, the reduction in filament length is more pronounced with the homodimeric *Pb*CPs, and the tentacle domain is at least partly dispensable for the *Pb*CP function.

**Fig. 7:**
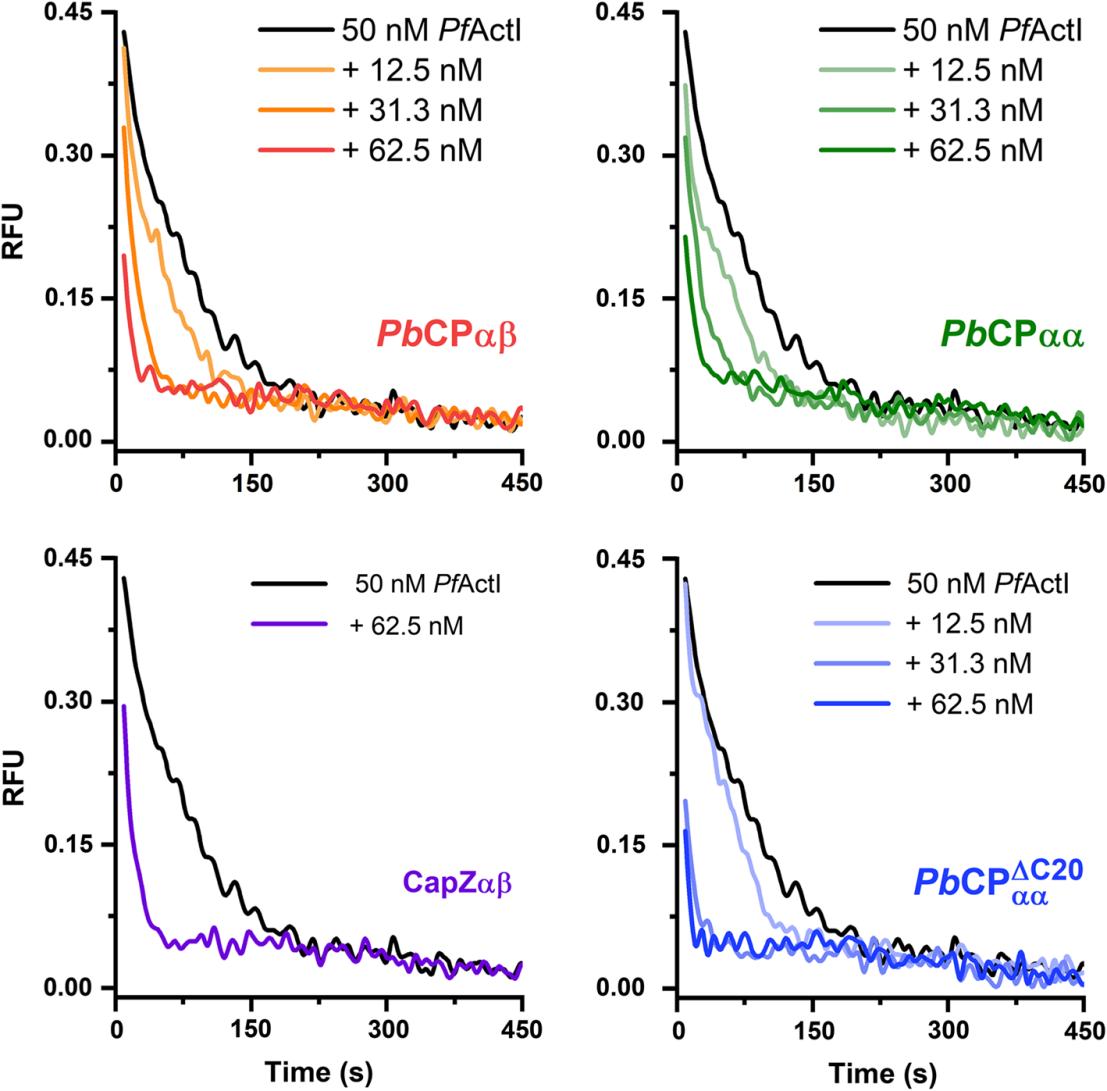
CPs facilitate *Pf*ActI depolymerization. **a** Dilution-induced depolymerization of *Pf*ActI filaments with various concentrations of *Pb*CPαβ, *Pb*CPαα, *Pb*CPαα^ΔC20^, and CapZαβ. The *Pf*ActI-only depolymerization curve is shared between measurements and the fluorescence levels are comparable. Concentrations shown are after F-buffer dilution. *RFU* relative fluorescent unit

*Pf*ActI filaments are highly dynamic and unstable [5–7] with high disassociation rates at both ends [9]. Upon dilution below their Cc_app_, filaments decompose rapidly, albeit slower than α-actin if filament end concentration is taken into account [6]. To investigate the barbed-end blocking efficiency of *Pb*CPs, we followed the disassembly of fluorescently labeled actin filaments. In contrast to canonical CPs [24], both homo- and heterodimeric *Pb*CPs facilitate the depolymerization of both *Pf*ActI and α-actin filaments (Figs. 7, S9). The increased velocity can be partly attributed to an increased filament end concentration [6], caused by shortening of the filaments (Fig. 6). However, this may not be the only explanation. Interestingly, CapZαβ also increased the rate of *Pf*ActI depolymerization (Fig. S9), suggesting that the inherent instability of *Pf*ActI filaments [8] also modulates the activity of actin regulators. This is in line with CapZαβ increasing the initial elongation rate of *Pf*ActI in the polymerization assays (Fig. 5a).

## Discussion

Owing to the intertwined and complementary nature of the canonical CPα and CPβ subunits, both are usually required to reconstitute a functional CP in vitro [37, 55]. Thus, the presence of a homodimeric CP in *Plasmodium* is surprising [23]. Sequence conservation mapped on *Plasmodium* CPα subunits reveals highly conserved residues located mainly at intersubunit interfaces in the *Pb*CPαα^ΔC20^ structure. This suggests that CPαα homodimers likely are conserved across the different *Plasmodium* species, as underlined by previous *Pf*CP complementation experiments [23]. So far, we have not been able to detect any evidence of CPββ homodimers, although the presence of such or CPα or CPβ monomers in the parasite cannot be ruled out. It is notable that *Drosophila melanogaster* CPαβ subunits are also encoded by single genes with independent in vivo functions implicated, but the protein still remains an obligate functional heterodimer [56, 57]. *Pb*CPαα is an example of an asymmetric homodimer, not very common among proteins [46]. The twofold rotational symmetry of the CP dimer is broken by the disrupted interdimer interface due to the steric occlusion of the stalk domains. The lack of similar structural complementarity in *Pb*CPαα^ΔC20^ as in canonical CP heterodimers must stem from the limitations set by the identical amino acid sequences of the subunits. While the protruding loop of the *Plasmodium-*specific insert does not impact the conserved fold of the cap structure, the helical backbone of the dimer is distorted by the presence of a C-terminal β-hairpin motif of *Pb*CPα_1_^ΔC20^. Interestingly, the previously identified C-terminal Ala270 degradation point of *Pb*CPαα [43] is located on the first β-sheet of this hairpin motif. This further supports our hypothesis that this cleavage may be biologically relevant by alleviating any residual steric incompatibilities upon homodimerization. The deviations from the homodimeric symmetry perturb the canonical binding sites for actin [39, 41], PIP_2_ [58], and other CP regulators [51, 52, 59]. In line with this, we could not identify any regulatory proteins for CP in the *Plasmodium* genome.

CPs characterized to date cap filament barbed ends through a highly conserved mechanism [27, 39, 41]. They play critical roles in regulating actin dynamics by increasing Cc, nucleating filament formation, and decreasing F-actin elongation and disassembly rates [24]. The actin binding of homodimeric *Pb*CP in the absence of canonical barbed-end binding motifs, however, challenges our current understanding of parasite actin filament capping. Despite large structural differences, homo- and heterodimeric *Pb*CPs affect filament kinetics of both parasite and canonical actins. Also *Pf*ActI differs greatly from α-actin in terms of filament structure [5, 7], dynamics [6, 8] and barbed-end electrostatics. Furthermore, C-terminally truncated *Pb*CPαα retains its ability to bind both β-actin [23] and *Pf*ActI filaments [43]. This implies that actin filament capping by *Pb*CPαα is independent of the α-tentacle and the basic patch. The *Plasmodium*-specific insert contains several highly conserved residues and could compensate for the missing conventional interaction points. The presence of two of such inserts and a non-canonical cap structure indicates an atypical barbed-end binding mode and maybe a distinct functional role for the CP homodimers, as compared to the canonical or parasite CP heterodimers [22, 23, 43].

Both homo- and heterodimeric *Pb*CPs increase the amount of *Pf*ActI present in the soluble fraction after pelleting the filaments [43]. Here, we show that this is due to filament shortening and not due to an increase in the steady-state G-actin concentration, thus explaining the inability of *Pb*CPs to raise the Cc_app_. While homo- and heterodimeric *Pb*CPs regulate actin kinetics in vitro in a similar manner, it seems that the homodimers may also be able to stabilize or sequester short, non-nucleating structures, perhaps lateral dimers, which are a significant species in *Pf*ActI at least in vitro [6]. Such a function could be facilitated by the presence of two *Plasmodium*-specific inserts in the homodimer. The sequestration of dimers would explain the increased steady-state and initial fluorescence levels in polymerization assays and the disappearance of filaments. It is possible that *Pb*CPs cannot completely block the barbed end or that the Cc for *Pf*ActI is equal at both filament ends, as proposed by a similar *k*_off_ at each end [9]. Subunit exchange at the CP-bound barbed end would suggest a “wobbly” capping behavior, found in processive barbed-end cappers [24], such as formins [54]. The existence of flexible domains in *Pb*CPs and their positive effect on filament assembly correspond well with the properties of a wobbly capper.

*Pb*CPs appear to lack prominent nucleating capabilities. Thus, the acceleration of actin depolymerization by *Pb*CPs could be explained by severing. However, such mode of action has been disproven for CapZαβ [50, 60, 61]. Another explanation could stem from the innate fragility of *Pf*ActI filaments and their propensity to fragmentation [8]. Collision of *Pb*CPs with the barbed end could promote severing events and depolymerization instead of forming a stable cap structure. After such an interaction they could dissociate or remain weakly bound to the filaments. This hypothesis is supported by the inability of CapZαβ to co-sediment with *Pf*ActI filaments, yet still increasing the soluble actin fraction [43] and reducing the average filament length.

Intracellular eukaryotic parasites undergo reductive genome evolution towards a streamlined set of proteins [62]. A significantly reduced repertoire of ABPs in *Plasmodium* has given rise to atypical functions for many of the proteins [13]. One outcome of this adaptation may be the emergence of a homodimeric form of CP [23, 43]. Motility and host cell invasion of *Plasmodium* parasites rely on rapid turnover of short, unbranched filaments [63]. As *Pf*ActI is less polymerization competent and less stable than canonical filaments [5], stringent F-actin capping might be too restrictive for the parasite motility requirements. Wobbly capping by *Pb*CPs that promotes turnover and reduces the length of *Pf*ActI filaments may be suited for the parasitic lifestyle. Despite the functional redundancy in vitro, *Pb*CPαα and *Pb*CPαβ cannot complement each other in vivo [23]. Thus, the adaptation of the homo- and heterodimeric forms to the cellular niches of their respective insect and vertebrate hosts stems from their inherent structural differences.

Heterodimeric *Pb*CP has two different interfaces with the last two actin protomers at the barbed end, which could enable it to selectively bind to filaments but not to symmetric dimers. *Pb*CPαβ is required for sporozoite motility and salivary gland invasion [22]. The quick utilization of actin monomers during these processes is possibly enabled by the reduced dimer-binding capability of *Pb*CPαβ as compared to *Pb*CPαα. The β-tentacle of CapZαβ is crucial for the controlled growth of a branched actin network [41]. In the absence of the Arp2/3 branching complex and nucleation promoting factors [12], *Plasmodium* only has short linear actin filaments. Thus, without the need for a such control element, *Pb*CPβ has evolved a divergent C-terminal domain. While the conserved hydrophobic residues implied in a canonical barbed-end interaction are still present, it also contains additional stretches of residues, possibly enabling looser capping of the filaments.

Homodimeric *Pb*CP contains two nearly identical interaction surfaces and might thus be more suited for binding two actin monomers, like the active form of gelsolin [64]. As gelsolin is missing from the *Plasmodium* genome [42], a similar activity could have evolved in *Pb*CPαα. The proposed dimer binding by *Pb*CPαα could play a role in restricting the number of filaments during the non-motile erythrocytic stages, during which the homodimers are indispensable [23]. In the absence of the Arp2/3 complex or gelsolin, formin remains the only characterized actin nucleator in *Plasmodium* [17]. Formins are localized at the moving junction (MJ) during erythrocytic invasion by non-motile merozoites [65]. As the correct formation and stringent cytoskeletal control of the MJ is critical [66], the wobbly capper formins could partially compensate for the phenotypic lack of *Pb*CPβ [22]. The existence of an actin:CP:formin ternary complex [67] supports this hypothesis.

Based on our kinetic data, *Pb*CPs have at least an order of magnitude lower affinity towards *Pf*ActI filaments than CapZαβ to α-actin. Left unregulated, canonical CPs would constantly cap actin filaments, completely arresting cell motility [32]. Due to the reduced set of CP regulators, *Pb*CPs might have evolved a low-affinity wobbly capping behavior, allowing filament turnover without the need for external modulating agents. Without such regulators in the genome, canonical regulator interaction sites are also missing. The self-sufficiency of *Pb*CP regulation is further underlined by the reduced intersubunit interface area of *Pb*CPαα^ΔC20^ together with the low thermal stability of the *Pb*CPs, and a broader unfolding peak of *Pb*CPαβ [43]. These may reflect subtle temperature-induced structural changes, possibly relevant for the transitioning of the parasite between the cold-blooded insect host where *Pb*CPβ is upregulated [68], and the warm-blooded vertebrate host where the *Pb*CPαα form is relevant [23].

## Concluding remarks

CPs are essential regulators of the cytoskeleton and conserved among metazoans as heterodimeric proteins, which are important, e.g., for lamellipodial protrusions at the leading edge of crawling cells [26]. Apicomplexan parasites display fast gliding motility without changes in cell shape [69]. It seems that due to different cytoskeletal requirements in different lifecycle stages, these parasites have adopted a homodimeric form of CP [43] as part of their limited repertoire of ABPs [13]. In contrast to canonical CPs [24], homo- and heterodimeric *Pb*CPs facilitate the rapid turnover of the dynamic *Plasmodium* actin filaments supporting the unusual actomyosin machinery of the parasite.

## Materials and methods

### Protein expression and purification

*Pb*CPαβ, *Pb*CPαα, *Pb*CPαα^ΔC20^, CapZαβ, α-actin, and *Pf*ActI were prepared as described previously [6, 43].

### Crystallization and structure determination

*Pb*CPαα^ΔC20^ was crystallized at 4 °C using the vapor diffusion method. Crystals were grown from a 1:1 drop ratio of 10–15 mg/mL protein and precipitant [100 mM MES pH 6.5, 100–150 mM tri-ammonium citrate, 10–12% (w/v) PEG 20,000, and 0.5 M NDSB-195 (Hampton, US)] and subsequently improved by iterative micro-seeding. The crystals were soaked for 1–2 min in 1 M NaBr before flash freezing in 15% (v/v) glycerol. Diffraction datasets were collected on the I04-1 beamline at Diamond Light Source (Didcot, UK) using a Pilatus 6 M detector and 0.916 Å beam wavelength. Datasets from multiple crystals were integrated, scaled, and merged using DIALS [70] in the xia2 pipeline [71]. The initial experimental phases and polyalanine main-chain trace were obtained from SHELX [72]. The structure was refined using the CCP4 package [73] and Phenix [74], with iterative rebuilding in Coot [75].

### Structural bioinformatics

Model of *Pb*CPαα^ΔC20^ capped *Pf*ActI filament was prepared by aligning 5 protomers of *Pf*ActI filament (PDB ID: 5OGW [7]) and *Pb*CPαα^ΔC20^ to the CapZαβ capped barbed end of the Arp1 filament in dynactin (PDB ID: 6F1T [48]) using TM-align [76]. Gaps in the structure of *Pb*CPαα^ΔC20^ were modeled in using SWISS-MODEL. The relative orientation of *Pb*CPαα^ΔC20^ was refined with RosettaDock [77], while residues involved in the interface were energy minimized with UCSF Chimera. Electrostatic potential surfaces were calculated using APBS [78]. EMBOSS Matcher [79] was used for local sequence alignments and ESPript [80] for their visualization. 21 non-redundant *Plasmodium* CPα sequences from PlasmoDB were analyzed, with conservation score subsequently mapped on the structure of *Pb*CPαα^ΔC20^ using ConSurf [81]. CapZαβ was assessed similarly, using automatically retrieved homolog sequences. Details of the surface area and r.m.s.d. calculations are found in Supporting Information. Database searches against homologs and motifs were carried out using the PlasmoDB database [82], ScanProsite [83], MyHits [84], and PATTINPROT [85].

### Small-angle X-ray scattering

SEC-SAXS data of *Pb*CPαα and *Pb*CPαβ, at respective concentrations of 12 and 5.8 mg/mL, were collected on the B21 beamline at Diamond Light Source (Didcot, UK). Data processing, ab initio DAMMIN [86] modeling, and visualization were carried out as previously described [43]. Initial models of the homodimers were prepared by extending the crystal structure of *Pb*CPαα^ΔC20^ using SWISS-MODEL [87]. The tentacle domain of *Pb*CPα_1_ was modeled using EOM [88]. A model of *Pb*CPαβ was assembled from the modeled *Pb*CPα_2_ subunit and I-TASSER [89] modeled *Pb*CPβ monomer using the structure of dynactin-bound *S. scrofa* CapZαβ (PDB ID: 6F1T [48]) as a template. The models were corrected against major steric clashes using Coot [74] and energy minimized using UCSF Chimera [90]. The *Pb*CP models were split into domains and refined with normal mode analysis using SREFLEX [91].

### Actin-CP interaction assays

Fluorescence-based polymerization assays were carried out in triplicate and analyzed as previously described [6] with minor modifications. Cc_app_ was determined using a dilution series prepared from 10 µM actin polymerized together with 10 µM CP for *Pf*ActI, and 0.4 µM CP for α-actin. 4 µM total actin concentration was kept in the polymerization assays. 0.5 µM polymerized unlabeled *Pf*ActI was used as nuclei in the preseeded polymerization assays. In the depolymerization assays 4 µM *Pf*ActI was polymerized together with the CPs for 16 h at 20 °C before dilution in F-buffer below Cc_app_. The polymerization data were despiked before averaging. The depolymerization data were shifted to match the baseline of the actin control. No pointed end cappers were used in the assays to limit subunit exchange to the barbed end.

### Electron microscopy

20 µM *Pf*ActI was polymerized with or without 25 µM CPs in F-buffer at 20 °C for 16 h. Prior sample application, the carbon-coated 300-mesh copper grids (Gilder, US) were glow-discharged for 1 min. After diluting *Pf*ActI to 1 µM with F-buffer, grids were immediately prepared using a previously reported protocol [8]. Negative stained grids were imaged using a Tecnai G2 Spirit microscope (FEI, US) operated at 120 kV with a pixel size of 0.592 nm. Micrographs were analyzed using the Ridge Detection plugin [92] of Fiji [93]. Particles smaller than 12 nm were excluded to omit bias caused by any unbound CP. Mann–Whitney tests were carried out using OriginPro (OriginLab, US).

### Supplementary Information

Below is the link to the electronic supplementary material.

Supplementary file1 (PDF 27796 kb)

## Data Availability

The atomic coordinates and structure factors of *Pb*CPαα^ΔC20^ have been deposited in the Protein Data Bank, https://www.ebi.ac.uk/pdbe/ (PDB ID: 7A0H).
