# Supplementary material for: Structure and function of an atypical homodimeric actin capping protein from the malaria parasite
Source: Cell Mol Life Sci. 2022 Feb 8;79(2):125. doi: 10.1007/s00018-021-04032-0 (PMC8821504; doi:10.1007/s00018-021-04032-0)
Supplement: Supplementary file 1 — Supplementary file1 (PDF 27796 kb) [file 18_2021_4032_MOESM1_ESM.pdf]

SUPPLEMENTARY INFORMATION

Structure of an atypical homodimeric actin capping protein from the malaria parasite

Ábris Ádám Bendes[1], Petri Kursula[1,2], and Inari Kursula[1,2*]

[1] Biocenter Oulu and Faculty of Biochemistry and Molecular Medicine, University of Oulu, Oulu, Finland

[2] Department of Biomedicine, University of Bergen, Bergen, Norway

[*] Corresponding author, e-mail address: inari.kursula@uib.no (IK)

SUPPLEMENTARY METHODS

Sequence alignment

Protein sequence alignments utilizing structural information were carried out using T-COFFEE [1]. The alignments were visualized using ESPript [2]. Secondary structure and residue contact information were extracted from the SWISS-MODEL [3] extended *Pb*CPαα$^{\Delta C20}$ structure and the I-TASSER [4] homology model of *Pb*CPβ for the CPα and CPβ subunits, respectively. ConSurf [5] conservation was calculated as discussed in Materials and Methods. Residues of canonical CPs involved in characterized interactions are reviewed in Eckert et al. [6]. Residues involved in CP binding through forming the acidic and hydrophobic patch in actin or Arp1 are taken from literature sources [7, 8].

Electrostatic potential calculation

The *Pb*CPαβ-capped barbed end model was assembled by substituting the capped Arp1 filament in 6F1T [9] with *Pf*ActI protomers (PDB ID: 5OGW [10]). The CapZαβ was replaced with the NMA-refined *Pb*CPαβ model (Fig. 3d). The *Pb*CPαβ-capped barbed end was refined similar to the *Pb*CPαα$^{\Delta C20}$-capped barbed end as detailed in Materials and Methods. The prepared model was subsequently aligned to the structure of bovine β-actin filament capped with murine CapZαβ (PDB ID: 7PDZ [11]) by superimposing only the actin protomers. The protein chains were aligned using TM-align [12]. Electrostatic potential surfaces of the were calculated using APBS [13].

Surface area calculation

Gaps in the *Pb*CPαα$^{\Delta C20}$ structure were extended using SWISS-MODEL prior to analysis. The dimer interface and surface areas of the CPs were calculated using the jsPISA server [14]. The total accessible surface area (ASA) for *Pb*CPαα$^{\Delta C20}$ and CapZαβ (PDB ID: 6F1T) were calculated using PyMOL [15].

Root-mean-square deviation calculation

Sequence-independent root-mean-square (r.m.s.d.) calculations of the CP isoforms and their domains [PDB ID: 1IZN, 4AKR [6], 6F1T, and 7A0H (this work)] were carried out using TM-align. Gaps in the *Pb*CPαα$^{\Delta C20}$ structure were extended using SWISS-MODEL prior to analysis.

Critical concentration assay under Ca^{2+} conditions

Human full-length gelsolin was purchased from Cytoskeleton (US). Cc assay with 10 µM *Pf*ActI was prepared similar to as described in Materials and Methods with the difference that EGTA was omitted from the buffers ensuring that the added 0.4 µM gelsolin remained functional.

Polymerization assay with α-actin

Assays were prepared with 4 µM total α-actin concentration similar to as described in Materials and Methods with the difference that 2 µM polymerized unlabeled α-actin was used as nuclei in the preseeded assays.

Depolymerization assay with preformed actin filaments

Assays were prepared similar to as described in Materials and Methods with the difference that actins were polymerized alone for 16 h at 20°C beforehand. CPs were incubated for 5 min at 20°C with 4-5 µM polymerized *Pf*ActI or α-actin before dilution in F-buffer below Cc_{app}.

SUPPLEMENTARY TABLES

Table S1 Data collection and refinement statistics.

Data collection	*Pb*CPαα$^{\Delta C20}$
Wavelength (Å)	0.916
Resolution range (Å)	79.65-2.22 (2.30-2.22)†
Space group	C 1 2 1
Cell dimensions	
a, b, c (Å)	164.49, 35.17, 115.44
α, β, γ (°)	90.00, 104.44, 90.00
No. of reflections	2516659 (51165)
Unique‡	61855 (6109)
Multiplicity	40.7 (8.4)
Completeness (%)	99.24 (95.57)
⟨I/σ(I)⟩	10.62 (0.45)
Wilson B-factor (Å^2)	49.65
R_{merge}	0.260 (4.37)
R_{pim}	0.0395 (1.57)
$CC_{1/2}$	0.999 (0.29)
Refinement	
Resolution range (Å)	74.20-2.22 (2.25-2.22)
No. of reflections	61419 (2419)
R_{work}	0.207 (0.420)
R_{free}§	0.257 (0.428)
No. of atoms	
Protein	4432
Ligand/ion	61
Solvent	149
r.m.s.d.	
Bond lengths (Å)	0.016
Bond angles (°)	1.47
B-factors (Å^2)	
Protein	107.4
Ligand/ion	93.0
Solvent	64.6
Ramachandran plot (%)	
Favored	96.42
Allowed	3.58
Outliers	0.00
Rotamer outliers (%)	0.59

† Data in parentheses represent the last resolution shell

‡ Calculated from unmerged Friedel pairs

§ R_{free} was calculated from a 5% test set

Table S2 Surface and interface areas of the CP dimers. Sarcomeric, cytoplasmic, and dynactin-bound CP isoforms are represented by chicken CapZαβ (*Gg*CapZαβ, PDB ID: 1IZN), slime mold CP (Cap32/34, PDB ID: 4AKR), and pig CapZαβ (*Ss*CapZαβ, PDB ID: 6F1T), respectively. ASA: accessible surface area, BSA: buried surface area, HB: hydrogen bonds, SB: salt bridges.

	*Gg*CapZαβ	Cap32/34	*Ss*CapZαβ	*Pb*CPαα$^{\Delta C20}$
Subunit ASA (Å^2)	17200 / 16900	15400 / 14700	16800 / 16600	17400 / 17200
Subunit BSA (Å^2)	3900 / 3700	3700 / 3500	3700 / 3600	2300 / 2400
Total ASA (Å^2)	26600	23000	26800	30600
Total BSA (Å^2)	7600	7200	7300	4700
HB / SB	45 / 6	42 / 9	44 / 10	18 / 0

Table S3 R.m.s.d. values of different CP dimers superimposed on each other. Sarcomeric, cytoplasmic, and dynactin-bound CP isoforms are represented by *Gg*CapZαβ (PDB ID: 1IZN), Cap32/34 (PDB ID: 4AKR), and *Ss*CapZαβ (PDB ID: 6F1T), respectively. The r.m.s.d. values are given in Å and colored from green to red relative to the values across Tables S3-5

CP dimers	*Gg*CapZαβ	Cap32/34	*Ss*CapZαβ	*Pb*CPαα$^{\Delta C20}$
*Gg*CapZαβ	0.0	1.8	3.0	4.9
Cap32/34	1.8	0.0	2.3	5.0
*Ss*CapZαβ	3.0	2.3	0.0	4.6
*Pb*CPαα$^{\Delta C20}$	4.9	5.0	4.6	0.0

Table S4 R.m.s.d. values of different CP subunits superimposed on each other. Sarcomeric, cytoplasmic, and dynactin-bound CP isoforms are represented by *Gg*CapZαβ (PDB ID: 1IZN), Cap32/34 (Denoting subunits β and α, respectively. PDB ID: 4AKR), and *Ss*CapZαβ (PDB ID: 6F1T), respectively. The r.m.s.d. values are given in Å and colored from green to red relative to the values across Tables S3-5

CP subunits	*Gg*CapZα	*Gg*CapZβ	Cap34	Cap32	*Ss*CapZα	*Ss*CapZβ	*Pb*CP$\alpha_1^{\Delta C2}$	*Pb*CP$\alpha_2^{\Delta C2}$
*Gg*CapZα	0.0	4.4	1.7	3.8	2.3	4.7	2.9	3.5
*Gg*CapZβ	4.4	0.0	3.5	1.1	4.7	2.6	4.3	3.9
Cap34	1.7	3.5	0.0	3.4	2.2	4.5	2.9	3.3
Cap32	3.8	1.1	3.4	0.0	3.7	2.1	4.3	3.9
*Ss*CapZα	2.3	4.7	2.2	3.7	0.0	4.7	3.0	3.3
*Ss*CapZβ	4.7	2.6	4.5	2.1	4.7	0.0	4.6	4.2
*Pb*CP$\alpha_1^{\Delta C20}$	2.9	4.3	2.0	4.3	3.0	4.6	0.0	2.8
*Pb*CP$\alpha_2^{\Delta C20}$	3.5	3.9	3.3	3.9	3.3	4.2	2.8	0.0

Table S5 R.m.s.d. values of different CP domains superimposed on each other. Sarcomeric, cytoplasmic, and dynactin-bound CP isoforms are represented by *Gg*CapZαβ (PDB ID: 1IZN), Cap32/34 (Denoting subunits β and α, respectively. PDB ID: 4AKR), and *Ss*CapZαβ (PDB ID: 6F1T), respectively. The r.m.s.d. values are given in Å and colored from green to red relative to the values across Tables S3-5

CP domains		*Gg*CapZα	*Gg*CapZβ	Cap34	Cap32	*Ss*CapZα	*Ss*CapZβ	*Pb*CP$\alpha_1^{\Delta C2}$	*Pb*CP$\alpha_2^{\Delta C2}$
*Pb*CP$\alpha_1^{\Delta C20}$	Stalk	2.3	1.8	2.4	2.4	2.0	1.8	0.0	1.2
	Globule	2.0	2.8	2.0	2.8	2.4	2.9	0.0	1.4
	C. sheets	2.7	2.9	2.5	2.8	2.9	3.4	0.0	2.9
	H5 helix	1.3	2.0	1.2	1.5	2.1	2.0	0.0	1.0
*Pb*CP$\alpha_2^{\Delta C20}$	Stalk	2.6	2.0	2.5	1.8	2.5	2.0	1.2	0.0
	Globule	2.4	2.9	2.1	2.9	1.9	3.0	1.4	0.0
	C. sheets	2.8	2.8	2.8	2.5	2.6	2.7	2.9	0.0
	H5 helix	1.9	2.2	2.0	2.3	2.5	2.0	2.3	0.0

SUPPLEMENTARY FIGURES

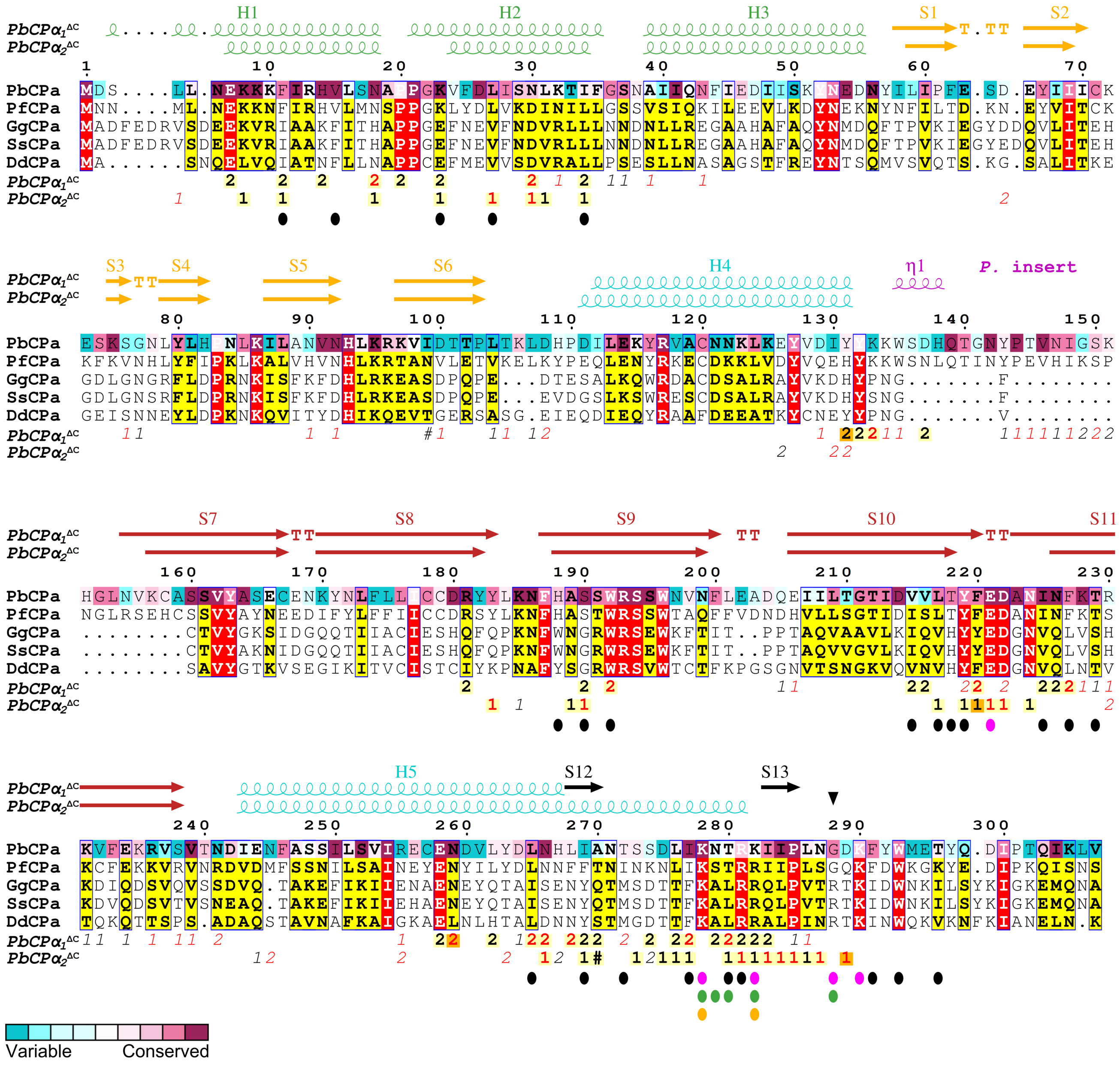

Fig. S1 Sequence conservation of CPα. The secondary structure elements of *Pb*CP$\alpha_1^{\Delta C20}$ and *Pb*CP$\alpha_2^{\Delta C20}$ are indicated on top of the alignment and colored as follows: green for the stalk, orange for the globule, cyan for the cap helices, purple for the *Plasmodium*-specific insert, red for the cap sheets, black for the C-terminus. The black triangle denotes the truncation point and start of the His$_6$-tag in *Pb*CPαα$^{\Delta C20}$ [16]. The aligned sequences (PbCPa: A0A509AR49, PfCPa: Q8I3I2, GgCPa: P13127, SsCPa: A0PFK5, DdCPa: P13022) were grouped and colored respective to a Risler matrix, using the ESPript convention. The PbCPa sequence is colored by ConSurf conservation scores (low: cyan, high: magenta) among *Plasmodium* CPα sequences. Intersubunit and crystallographic contacts are shown below the sequences using the *ESPript* convention (1: *Pb*CP$\alpha_1^{\Delta C20}$, 2: *Pb*CP$\alpha_2^{\Delta C20}$). Residues important for canonical intraheterodimer contacts, actin-, V-1/myotrophin-, and PIP$_2$-binding are indicated by black, magenta, green, and orange dots, respectively

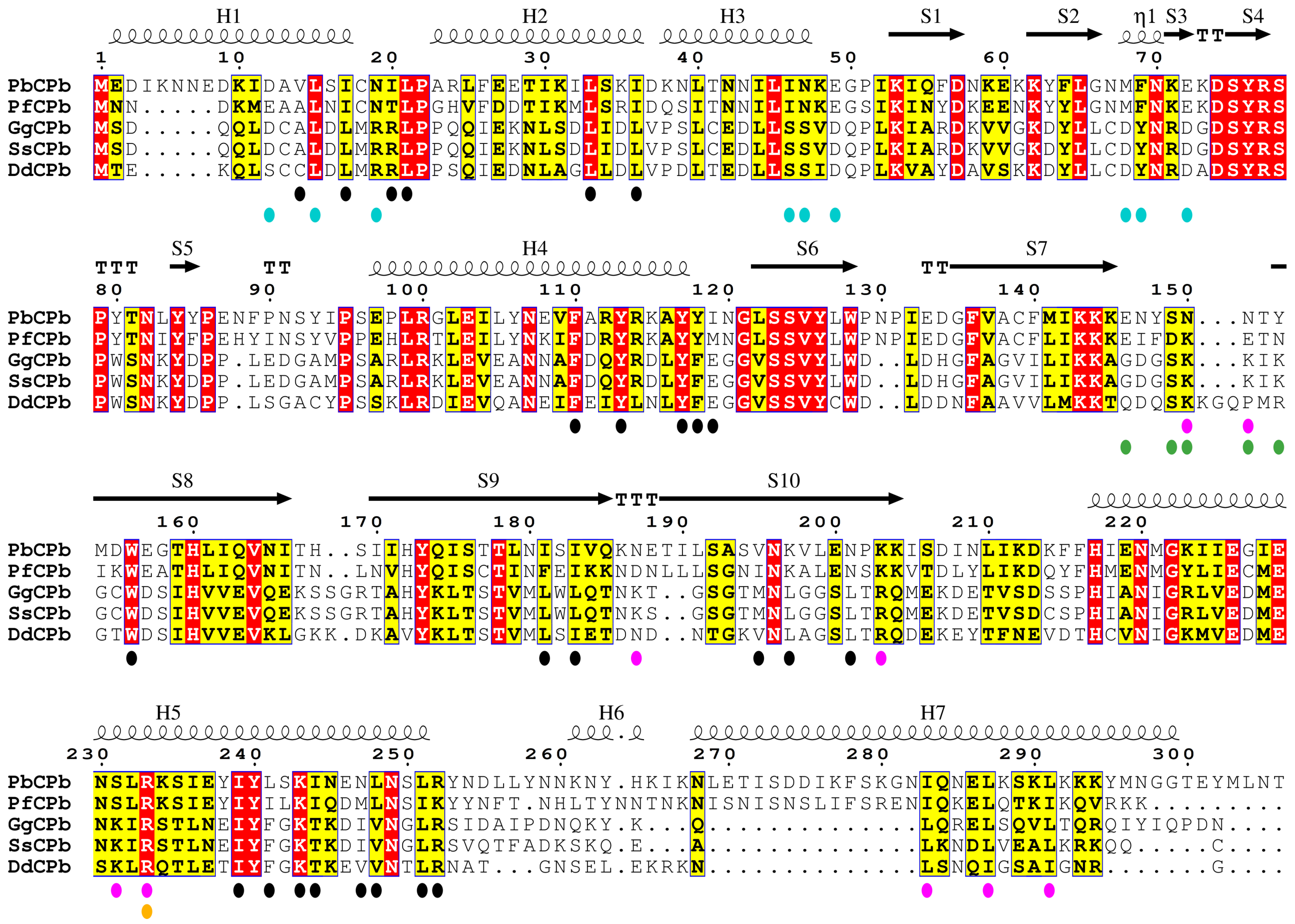

Fig. S2 Sequence conservation of CPβ. The secondary structure elements of *Pb*CPβ are indicated on top of the alignment. The aligned sequences (PbCPb: A0A509AQN8, PfCPb: Q8I3T2, GgCPb: P14315, SsCPb: A9XFX6, DdCPb: P13021) were grouped and colored respective to a Risler matrix, using the ESPript convention. Residues important for canonical intraheterodimer contacts, actin-, CARMIL-, V-1/myotrophin-, and PIP_2-binding are indicated by black, magenta, cyan, green, and orange dots, respectively

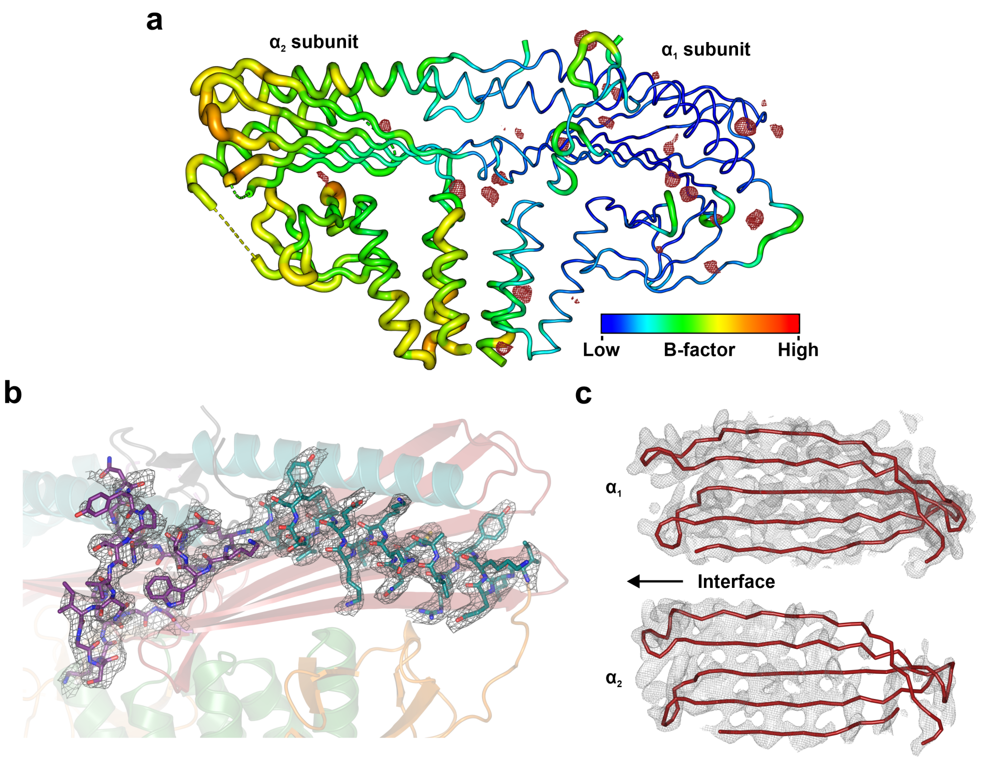

Fig. S3 Structural disorder in *Pb*CPαα$^{\Delta C20}$. **a** *Pb*CPαα$^{\Delta C20}$ dimer is displayed and colored according to C_α B-factors. The anomalous difference Fourier peaks of bromide atoms are shown with brown mesh contoured at 3 σ. Due to the disorder of *Pb*CPα$_2^{\Delta C20}$ interpretable electron density is missing for residues His104–Asp108 (between the globule and H4 helix), Lys134–Asn155 (the *Plasmodium*-specific insert), and Glu166–Asn169 (between the first and second central β-strands). **b** Electron density of the H4 helix and *Plasmodium*-specific insert of *Pb*CPα$_1^{\Delta C20}$. **c** Electron density of the cap sheets of *Pb*CPα$_1^{\Delta C20}$ (top) and *Pb*CPα$_2^{\Delta C20}$ (bottom). The 2F_o-F_c composite maps are shown with grey mesh contoured at 1 σ

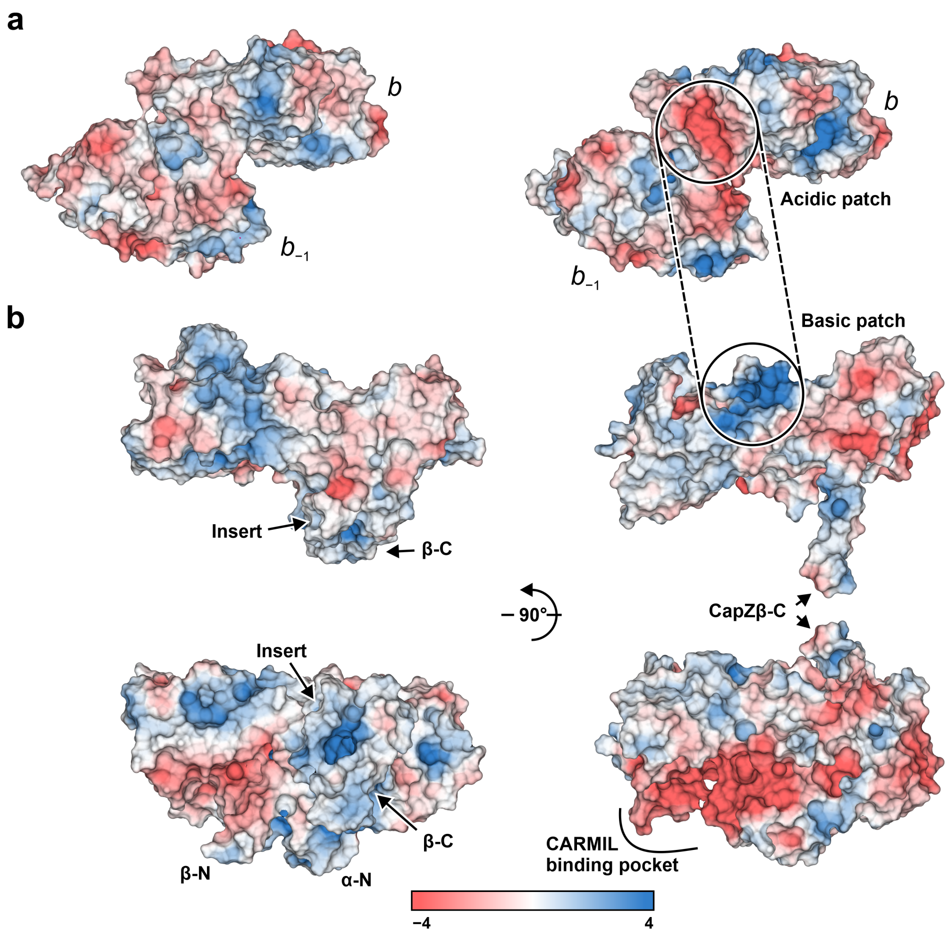

Fig. S4 Electrostatic potential surfaces at the barbed ends capped by heterodimeric CPs. **a** Last (*b*) and penultimate (b_{-1}) protomer of *Pf*ActI (left, PDB ID: 5OGW) and canonical β-actin filament (right, PDB ID: 7PDZ), in a CP-bound conformation. **b** Electrostatic potential surfaces of filament-bound *Pb*CPαβ (left) and CapZαβ (right, PDB ID: 7PDZ)

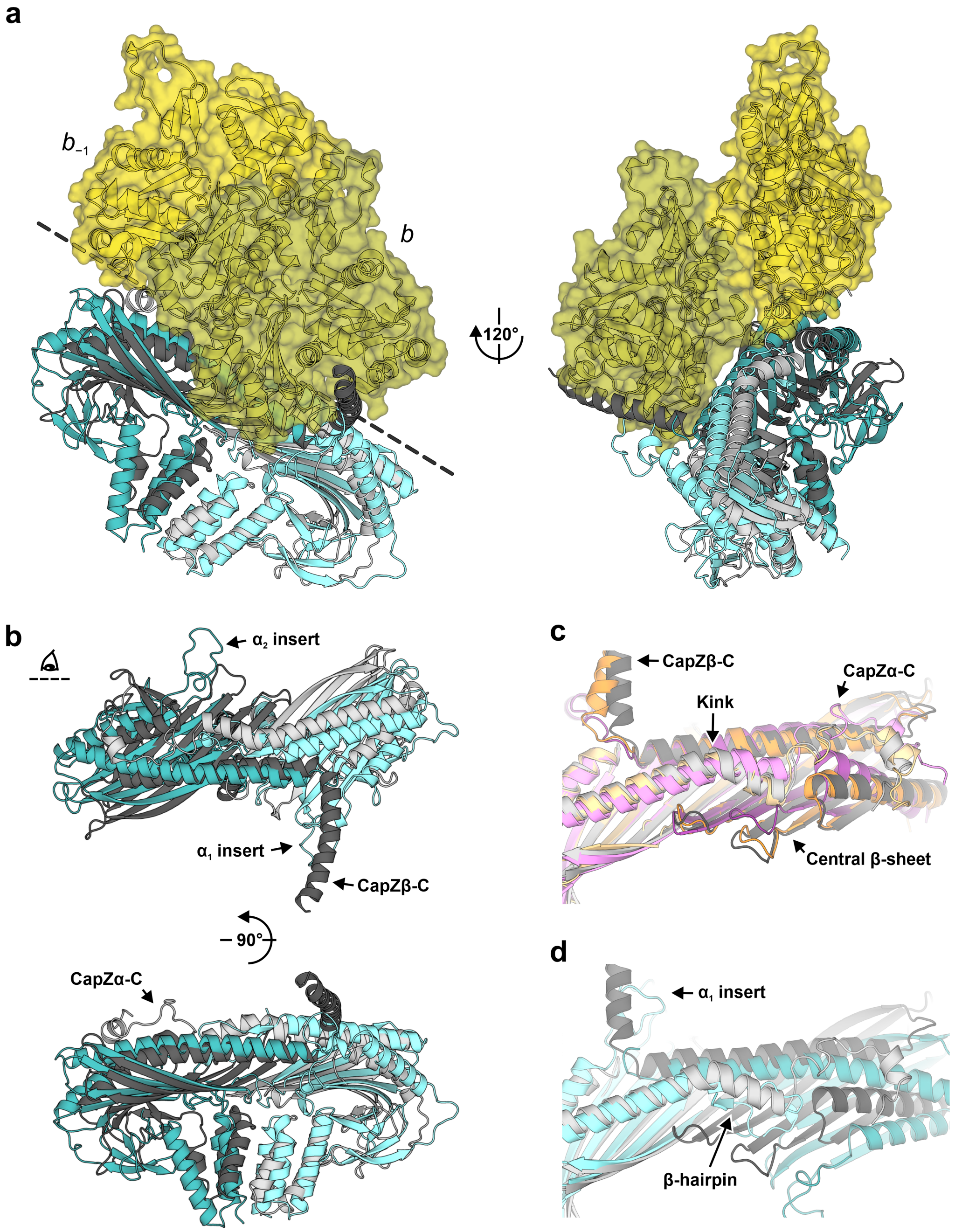

Fig. S5 Comparison of modelled and experimentally determined capped barbed end structures. **a** Last (*b*, green surface) and penultimate (*b*$_{-1}$, yellow surface) protomer of a β-actin filament capped by CapZαβ (CapZα and CapZβ are denoted by light and dark grey cartoons, respectively. PDB ID: 7PDZ). *Pb*CPαα$^{\Delta C20}$ is shown in the predicted relative orientation with *Pb*CPα$_1^{\Delta C20}$ and *Pb*CPα$_2^{\Delta C20}$ denoted by light and dark blue cartoons, respectively. **b** Relative orientation of CapZαβ (grey cartoon) and *Pb*CPαα$^{\Delta C20}$ (blue cartoon) at the barbed end. **c** Rearrangements in the CapZα H5 helix, α-tentacle and central β-sheets upon barbed end binding. Unbound, dynactin-bound, and β-actin-bound CapZαβ are represented by purple (PDB ID: 1IZN), orange (PDB ID: 6F1T), and grey cartoons, respectively. CPs were aligned by their CapZα subunits. **d** The H5 helixes of both *Pb*CPα$_1^{\Delta C20}$ (light blue cartoon) and β-actin-bound CapZα (light grey cartoon) are broken at similar locations and adopt comparable orientations after the kink

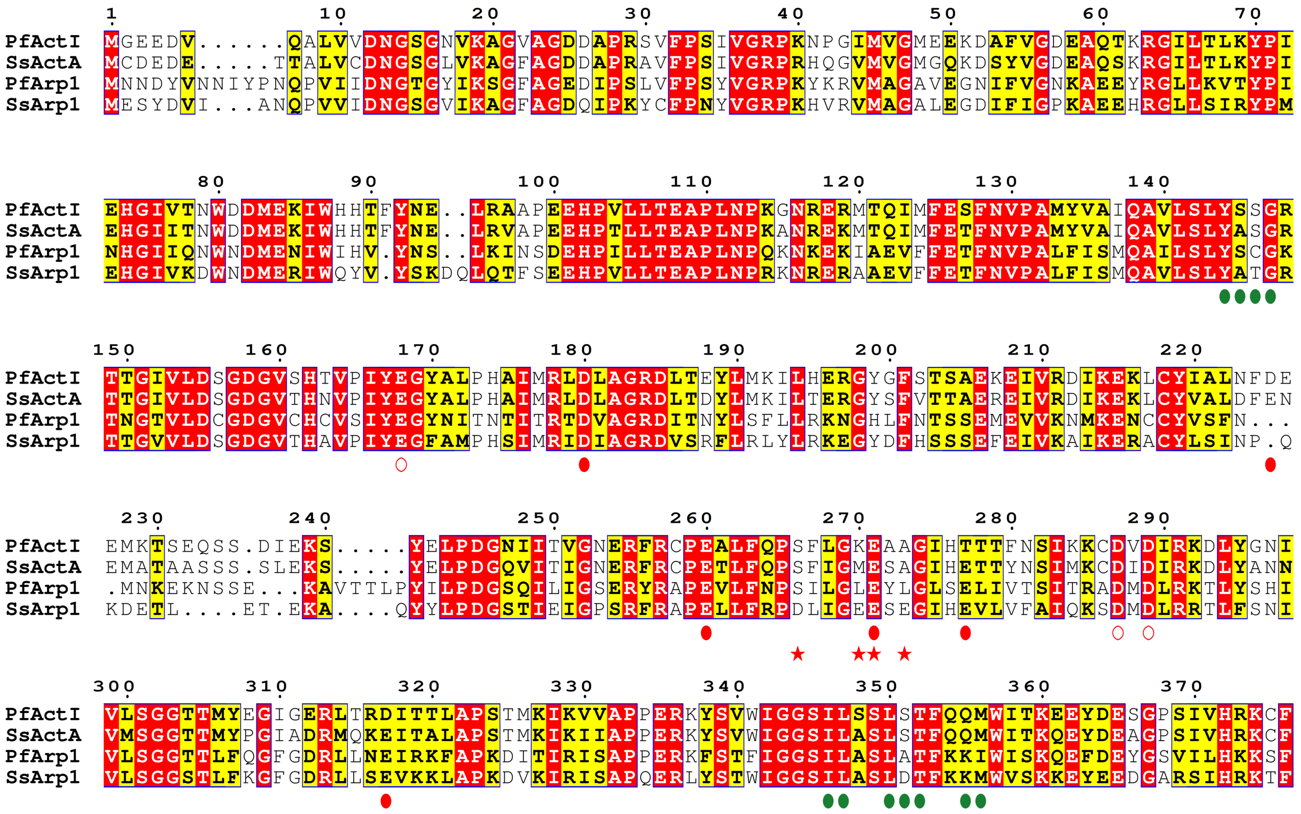

Fig. S6 Sequence conservation of actin and Arp1. The aligned sequences (PfActI: Q8I4X0, SsActA: P68137, PfArp1: Q8I2A2, SsArp1: F2Z5G5) were grouped and colored respective to a Risler matrix, using the ESPript convention. Red and green symbols show residues involved in forming the acidic or hydrophobic patch, respectively. Full dots or open circles denote residues on the last or penultimate actin filament protomer, respectively. Stars mark additional acidic residues on the last Arp1 monomer in the dynactin structure (PDB ID: 6F1T)

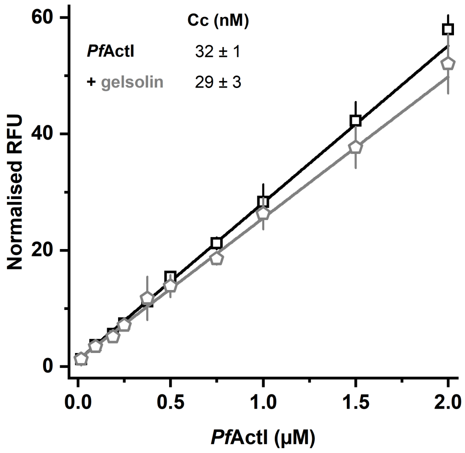

Fig. S7 Cc plot of *Pf*ActI filaments (open black squares) with gelsolin (open gray pentagon) under Ca^{2+} conditions. Note that the Cc_{app} of *Pf*ActI is different in Ca^{2+} vs. Mg^{2+} conditions [17], which explains the differences compared to Fig. 4a. Errors represent SD (n = 3). RFU = relative fluorescent unit, normalized to the lowest concentration

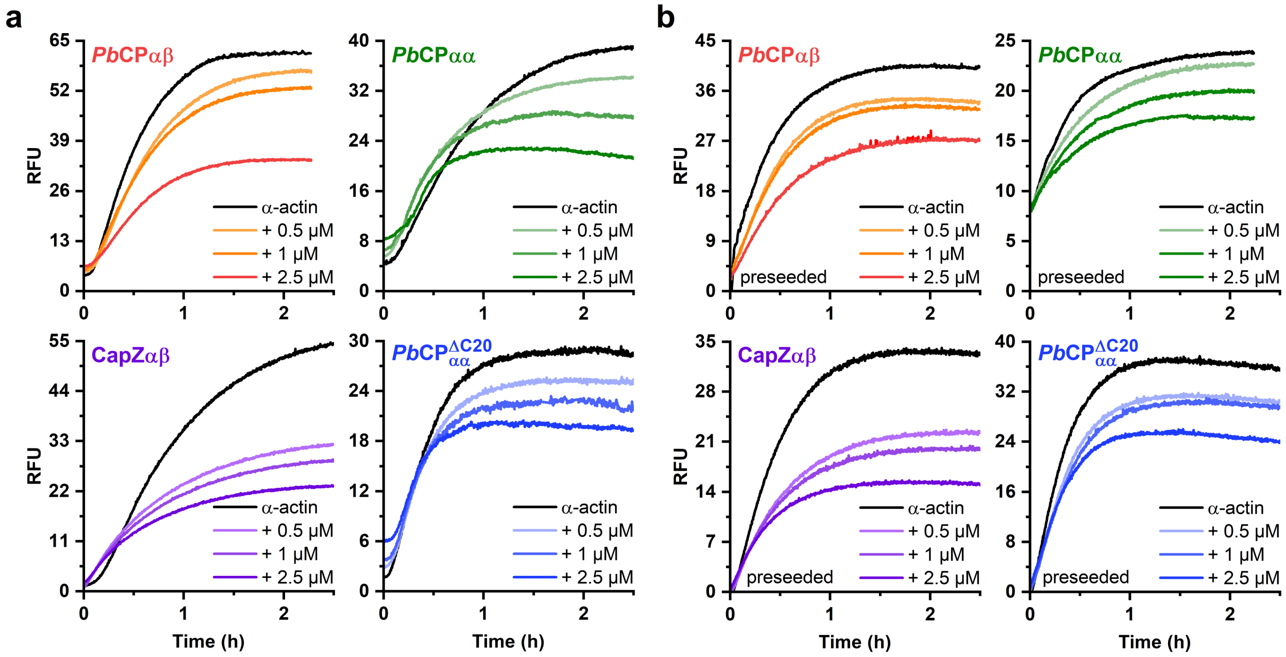

Fig. S8 Regulation of α-actin polymerization kinetics by *Pb*CPs. **a** Polymerization curves of α-actin in the absence and presence of increasing concentrations of *Pb*CPαβ, *Pb*CPαα, *Pb*CPαα$^{\Delta C20}$, and CapZαβ. **b** Polymerization of α-actin on capped, preformed homologous filaments in the absence and presence of increasing concentrations of *Pb*CPαβ, *Pb*CPαα, *Pb*CPαα$^{\Delta C20}$, and CapZαβ. RFU = relative fluorescent unit

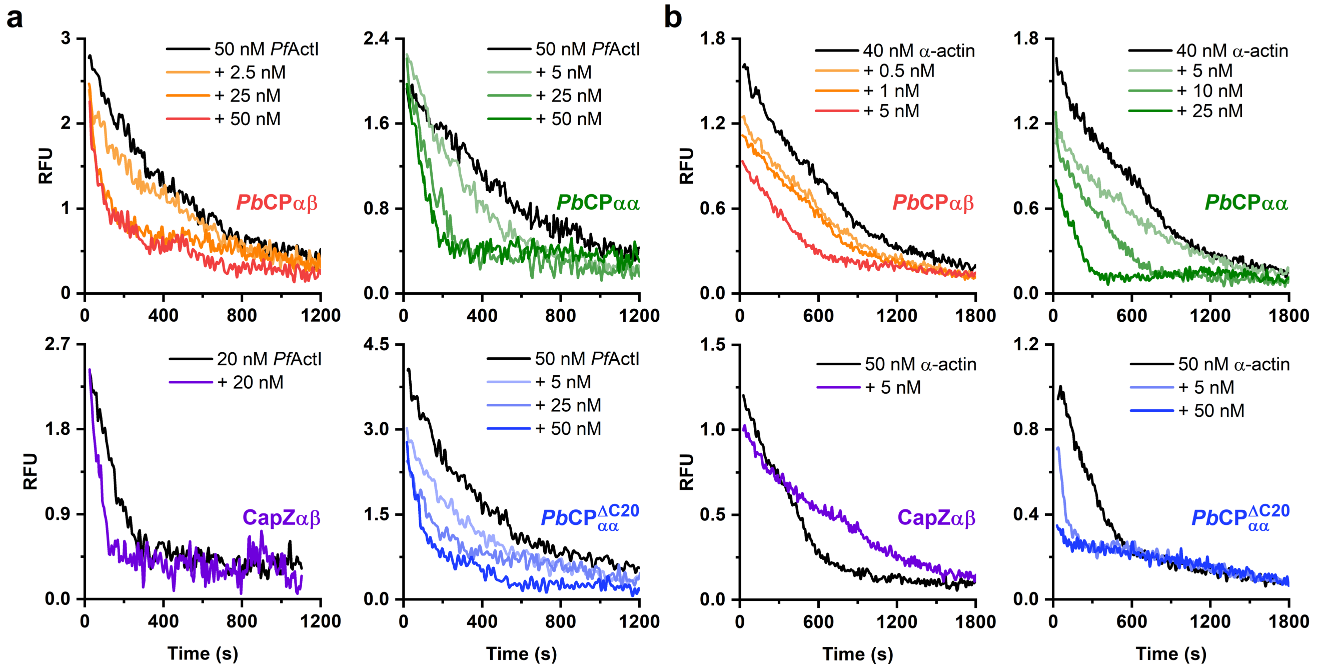

Fig. S9 *Pb*CPs facilitate actin filament depolymerization. **a** Disassembly of *Pf*ActI filaments with various concentrations of *Pb*CPαβ, *Pb*CPαα, *Pb*CPαα$^{\Delta C20}$, and CapZαβ. **b** Depolymerization of *Pb*CPαβ, *Pb*CPαα, *Pb*CPαα$^{\Delta C20}$, and CapZαβ capped α-actin filaments. Concentrations shown are after F-buffer dilution. RFU = relative fluorescent unit

SUPPLEMENTARY REFERENCES

1. Di Tommaso P, Moretti S, Xenarios I, et al (2011) T-Coffee: A web server for the multiple sequence alignment of protein and RNA sequences using structural information and homology extension. Nucleic Acids Research 39:. https://doi.org/10.1093/nar/gkr245
2. Robert X, Gouet P (2014) Deciphering key features in protein structures with the new ENDscript server. Nucleic Acids Research 42:. https://doi.org/10.1093/nar/gku316
3. Waterhouse A, Bertoni M, Bienert S, et al (2018) SWISS-MODEL: Homology modelling of protein structures and complexes. Nucleic Acids Research 46:W296–W303. https://doi.org/10.1093/nar/gky427
4. Yang J, Zhang Y (2015) I-TASSER server: New development for protein structure and function predictions. Nucleic Acids Research 43:W174–W181. https://doi.org/10.1093/nar/gkv342
5. Ashkenazy H, Abadi S, Martz E, et al (2016) ConSurf 2016: an improved methodology to estimate and visualize evolutionary conservation in macromolecules. Nucleic acids research. https://doi.org/10.1093/nar/gkw408
6. Eckert C, Goretzki A, Faberova M, Kollmar M (2012) Conservation and divergence between cytoplasmic and muscle-specific actin capping proteins: Insights from the crystal structure of cytoplasmic Cap32/34 from Dictyostelium discoideum. BMC Structural Biology 12:. https://doi.org/10.1186/1472-6807-12-12
7. Narita A, Takeda S, Yamashita A, Maéda Y (2006) Structural basis of actin filament capping at the barbed-end: A cryo-electron microscopy study. EMBO Journal 25:5626–5633. https://doi.org/10.1038/sj.emboj.7601395
8. Urnavicius L, Zhang K, Diamant AG, et al (2015) The structure of the dynactin complex and its interaction with dynein. Science 347:1441–1446. https://doi.org/10.1126/science.aaa4080
9. Urnavicius L, Lau CK, Elshenawy MM, et al (2018) Cryo-EM shows how dynactin recruits two dyneins for faster movement. Nature 554:202–206. https://doi.org/10.1038/nature25462
10. Pospich S, Kumpula EP, Von Der Ecken J, Vahokoski J, Kursula I, Raunser S (2017) Near-atomic structure of jasplakinolide-stabilized malaria parasite F-actin reveals the structural basis of filament instability. Proceedings of the National Academy of Sciences of the United States of America 114:10636–10641. https://doi.org/10.1073/pnas.1707506114
11. Funk J, Merino F, Schaks M, Rottner K, Raunser S, Bieling P (2021) A barbed end interference mechanism reveals how capping protein promotes nucleation in branched actin networks. Nature Communications 12:. https://doi.org/10.1038/s41467-021-25682-5
12. Zhang Y, Skolnick J (2005) TM-align: A protein structure alignment algorithm based on the TM-score. Nucleic Acids Research 33:2302–2309. https://doi.org/10.1093/nar/gki524
13. Jurrus E, Engel D, Star K, et al (2018) Improvements to the APBS biomolecular solvation software suite. Protein Science 27:112–128. https://doi.org/10.1002/pro.3280
14. Krissinel E (2015) Stock-based detection of protein oligomeric states in jsPISA. Nucleic Acids Research 43:W314–W319. https://doi.org/10.1093/nar/gkv314

15. Schrödinger L (2015) The PyMOL Molecular Graphics System, Version 2.0

16. Bendes ÁÁ, Chatterjee M, Götte B, Kursula P, Kursula I (2020) Functional homo- and heterodimeric actin capping proteins from the malaria parasite. Biochemical and Biophysical Research Communications 525:681–686. https://doi.org/10.1016/j.bbrc.2020.02.119

17. Kumpula EP, Pires I, Lasiwa D, et al (2017) Apicomplexan actin polymerization depends on nucleation. Scientific Reports 7:1–10. https://doi.org/10.1038/s41598-017-11330-w
